# Spirit of the English Periodicals, and Notices of English Medical Literature

**Published:** 1837-01-01

**Authors:** 


### Spirit of the English Periodicals, and Notices of English Medical Literature.

### Death.

There is a very good article on this subject of universal interest, in the eighth Part of the Cyclopædia of Anatomy and Physiology. There is no period of organic existence which does not fall within the sphere of medical investigation. We search with eager curiosity for the germ before it has been impregnated, and follow its subtle phases of development till it becomes a distinct object of sense. A living thing, it is continually under our inspection, every point of its physical structure is familiar to us, every function is curiously studied, and our great object is to prevent the occurrence of that event, which opens up new sources of scientific inquiry—death. Then come the anatomist and chemist, and how much of what we know respecting the living is drawn from our researches on the dead.

We do not intend to follow Dr. Symonds through his dance of death. Not that it is wearisome—far from that. But many of the steps are familiar, and the whole figure would be much too long. We will only select one or two of its parts, which may amuse or instruct the less severely practical portion of our readers.

Death, Dr. Symonds tells us, and we know it well, is a complex phenomenon. Absolutely, it is the total and permanent cessation of all the vital operations. But what the vulgar generally term death, is not death in so exact a sense. When consciousness, respiration, and the circulation cease, the individual is dead to the world. But some organic operations may go on for a season, the vermicular motions of the intestines may continue, the nails or beard will grow—and some vital properties are not yet extinguished, as the irritability of the muscular fibre, on the application of a stimulus.

The medical philosopher observes this distinction, and divides death into,

1. The cessation of certain functions of certain organs associated together to constitute a system ; in other words, the arrest of circulation, respiration, innervation. And—

2. The cessation of the vital properties of the tissues themselves, and of actions which go on between their particles, as nutrition and contraction.

This latter is molecular death—the former is christened systemic. A very little consideration of both will inform us:—1st, That molecular does not necessarily involve systemic death, unless the former is universal. 2dly, That when partial, as in mortification, the tendency of molecular to induce systemic death depends on the importance of the part to the whole. 3dly, That molecular death in one part can only induce the same change in another part, by means of its interference with one of the systemic functions. 4thly, That systemic death must necessarily be followed sooner or later by molecular death,—but that, 5thly, The reality of systemic death can only be proved with certainty by the occurrences pertaining to molecular death.

The imperfection of our philosophical language is always evident, when we speak of the more subtle vital phenomena. Dr. Symonds, for example, starts on the pursuit of molecular death, with the dogma that molecular life is constituted by two functions—nutrition and contraction. The term contraction is inadequate to express both the fact itself and our knowledge of it. If we examine the living muscle, we observe that it undergoes incessant changes which we term nutrition, and that it displays certain properties which we call irritability *or* contractility. If we turn to the osseous tissue, we see the same process of nutrition, but contractility in its obvious sense is lost. Even the animal basis of the bone fails to display the species of contraction that the muscle did, and the physiologist, finding distinctive phenomena, is compelled to employ terms which indifferently represent them. Sensible and insensible organic contractility are the exponents of vital actions, which are not contractions in its ordinary sense. But this is a digression, and we proceed.

Nutrition implies a mechanism or tissue of pores or infinitely minute tubes, the ingress and egress of fluid, and a certain quality of this fluid. Contraction, a fibrous arrangement of particles, in most animals, and in all a peculiar property called irritability or contractility. The violations of these conditions are necessarily followed by molecular death.

If nutrition be the property of the tissue, it follows logically, as in fact, that the destruction of the tissue is the destruction of nutrition. Molecular death *must* result from it.

If nutrition depends on the presence of a fluid, which we therefore call nutritive, the absence of the fluid must prevent nutrition. Such is the fact. Tie a hæmorrhoid so as to prevent blood getting into it, and it will inevitably die. That is mortification, and mortification is partial molecular death, its distinctive characters being the consequence of the persistence of life in other parts.

It is not so obvious at first sight, that retention of the fluid in the tissue must destroy nutrition. To make this a palpable consequence, another premiss is requisite. It is this, that nutrition implies not merely the presence of a nutritive material, but the more or less frequent renovation of it. That being granted, we no longer wonder at the retention of the fluid preventing nutrition, and so occasioning molecular death. It is a fact that it does so.

"Removal of the effete fluid is provided for in the Porifera by ejects; in the Polypifera by expulsion from the central cavity and by transpiration; in the Acalephæ by anal apertures; and in vascular animals by vessels especially appropriated to the purpose, by transpiration, and by various excretions. This condition of molecular life is less easily violated than those already spoken of, because the modes of fulfilling it are more numerous. This is equally true whether we speak of the simple animal forms, or of the tissues of the more complicated; mortification is less frequently the result of venous than of arterial obstruction. Unquestionably engurgescence and inflammation may ensue from the former, and may terminate in gangrene; but it is far more common for the part to be relieved by the excretion of various fluids, constituting hæmorrhage and dropsy, until new channels are found for the returning blood. Hence it appears that a redundance of fluid is less dangerous to organic structures than a deficiency."

That nutrition should be impaired or put a stop to by the depravation of the nutritive fluid is natural. That deterioration may be the result of imperfect respiration, of bad or scanty alimentation, or of insufficient or excessive excretion.

So much for molecular death from privation of nutrition. It results also from the deprivation of irritability, but it is questionable whether the two cases are analogous. The argument would be too long and too metaphysical for us just now; we therefore waive it. Irritability is at all events a primary function of the tissue, and its destruction implies an essential alteration of its state. Such is the matter of fact view. What is that alteration of state? We do not know. When the fibre of the muscle *ceases* to contract on the application of galvanism, we detect no change from its condition half an hour ago, when the irritability was perfect. The property appears to be exhausted, and that is all we ascertain. It is, indeed, though Dr. Symonds would seem to lean to a more flattering conclusion, an ultimate fact. Irritability is in the same category, quoad its nature, as sensation or volition. The former is the property of a fibre—the latter of the medullary matter. To be sure, one is more obviously physical than the others are; but, so far as our comprehension of all goes, they are ultimate facts. We only know that the brain sees, or smells, or wills—and we only know that the fibre contracts. The how and the why are beyond our reach. We may study the laws of the fact, though we do not know its essence or its causes. What tends to destroy irritability?

"Irritability may be destroyed by substances, either applied directly to the part or acting upon the general system. Thus, the fibres of the heart may be paralysed by a solution of opium injected into its cavities, or by essential oil of tobacco given by the mouth. Lightning annihilates the property all over the body. The motions of Infusoria may be arrested by a shock of galvanism, by solutions of opium and camphor, and by the vapour of sulphur. Arsenical preparations have a similar effect. The contractions of capillary vessels in the higher animals may be arrested by a certain description of injuries of the brain and spinal marrow."

Such is Dr. Symonds' account of molecular death. It is the deprivation of the molecular properties. That which is most striking as a molecular function is irritability—that which is most obvious in molecular death is the loss of it.

Systemic death, the destruction of the functions of the organs of assimilation, of the organ which circulates the nutritive fluid, of the organ which vitalises by aerating that fluid, and of the apparatus which feels and wills consciously or instinctively—in other words, the cessation of the operation of the heart, the lungs, and the nervous system, next comes under consideration. Dr. Symonds considers the causes of systemic death under the general head of syncope.

Of syncope he admits eleven forms, determined by its active causes. They are:—

Syncope by asphyxia.

Syncope by nervous lesions.

Syncope by injuries of the heart itself.

Syncope by injuries of other organs and tissue.

Syncope by mental emotion.

Syncope by hæmorrhage.

Syncope by poisons.

Syncope by cold and lightning.

Syncope by inanition.

Syncope by disease.

Syncope by old age.

A very coarse arrangement, and constituting, philosophically speaking, little better than a tangled coil. In examining these different sections we have repetitions of the same phenomena. Thus the syncope by disease or by poisons, may comprise every possible variety of syncope, considered in reference to its more immediate cause. It is clear that it has not been Dr. Symonds' object to analyse and reduce to their essential modes the varieties of death, but simply to consider the different forms of it as we see them on the surface. We should have preferred the former and the more scientific plan. We would have looked at death, as its commencement and its actual cause are to be found in the nervous system, or in the heart, or in the lungs. The variety of accidental modes tends only to distract and to confuse.

The sole point to which we shall advert in this long array of many kinds of syncope, is the syncope by nervous lesion. Our notions of the functions of the nervous system are unsettled. We have not yet arrived at an agreement upon what we do know. As we observed in our last number, men have been collecting the facts so busily, that they have not yet had leisure to reason very soundly on them. The comparative anatomy of the nervous system has not yet been properly worked out. The uncertainty in which we are at present with respect to the connexion between the brain and spinal marrow, and the functions of circulation and nutrition is to be lamented. Will the following quotation clear up the *alliance* of the brain and heart? We fear it does little more than add a smooth word to our vocabulary.

"But while there can be no question that all the organs are more or less related in the manner above indicated, it is not less evident that the connexion between some is of a far more intimate nature than between others. It is almost needless to instance the brain and the stomach, the brain, spinal marrow, and the heart, the heart and every part of the system, &c. &c. By overlooking the sympathetic relation between the brain and the heart, Bichat fancied that when he had proved the functional independence of the latter organ, he was compelled to search in some third part for the link between the death of the one and that of the other. It cannot be denied that in a large proportion of cases, the syncope which follows lesions of the cerebro-spinal system, is not a direct consequence, and that there is an intermediate suppression of the function of the lungs,—that in other words the syncope is the effect of asphyxia. (see Asphyxia.) It is somewhat remarkable that the illustrious physiologist just mentioned should have forgotten certain pathological facts which afford convincing evidence that cerebral injury may produce death without developing the phenomena of asphyxia; the 'apoplexie foudroyante,' for example, and the concussion of a blow or a fall. Nor is it less surprising that in his numerous experiments upon animals he should not have noticed what was afterwards fully demonstrated by Legallois and W. Philip, that both the heart and the capillaries may be immediately paralysed by violence done to the brain and spinal marrow. It must be remembered, however, that this result is much affected both by the extent and by the nature of the injury. Thus the brain may be sliced and the spinal cord divided, with no other influence upon the circulation than that which depends upon the interference with the respiratory actions; but laceration or crushing of the cerebral matter is immediately felt by the heart and capillaries. In these cases the circulation ceases, not because the cerebro-spinal axis takes any part in that function, but because it is connected with the heart in the same manner as we have stated that all the parts of the body are more or less connected,—in bonds of *alliance* though not of *dependence*. We have reason, however, to believe that the intimacy of the alliance between the brain and the heart is scarcely equalled by that of any other organs in the system."

We pass to the signs of approaching death. They possess a melancholy interest to the physician. To the vulgar they have always been matters of high import. The mystery that surrounds the future state of the *body*—its equally mystical connexion with the soul—have led men in all ages to attach an importance to the act of dying, which, philosophically, perhaps, it does not merit. The sad sarcasm of Rochefoucault is probably too true—that "most men die because they cannot help it," and just as such persons may be supposed to do. When consciousness is retained late in individuals of an intellectual character, there is a majesty in death even beyond what life can offer. Who does not recollect the imperial death of Trajan? "Raise me." An Emperor should die standing." Who can smile at the concluding moments of Rabelais? His last word a joke. Nowhere are the sweetest traits of humanity so conspicuous as on the dying man's bed—nowhere are the furies which Providence has made to dwell in the bosoms of the bad, so potent and so hideous. Yet, after all, the practical physician must confess, that the instances in which intellectual vigour does really remain to the last moments of existence are comparatively few. He must own too with a sigh, that the dreams of retribution on the death bed, are too frequently dreams only; and that the good and bad, the sinner and the sage, often die in precisely the same manner—the manifestations both of intellect and conscience being regulated rather by the nature of the malady, and by the *mode* of death, than by moral reflections on past life and conduct.

"The delirium of the dying is often of a most interesting character, and resembles dreaming more than any other form of derangement that has fallen under our notice. The ideas are derived less from present perceptions than in insanity, and yet are more suggested by external circumstances than in the delirium of fever and phrenitis. Thus the sight of a bystander often suggests the image of a friend long departed, in which character the moribund man addresses him, and talks earnestly of persons, scenes, and events belonging to a former period of his history as if still present. The vivified conceptions are generally derived from subjects which, either in his speculative pursuits, or in the business of life, have principally occupied his thoughts. The last words of Dr. Armstrong were addressed to an imaginary patient upon whom he was impressing the necessity of attention to the state of the digestive organs. We have heard that a great legal officer not long deceased, having raised himself for a moment from his couch, said with his wonted dignity, 'Gentlemen of the Jury, you will find,'—and then fell back on his pillow and expired. The visual conceptions reproduced in some minds often appear to have been derived from poetical reading. We remember hearing a young man, who had been but little conversant with any but civic scenes, discourse most eloquently a short time before death, of 'sylvan glen and bosky dell,' purling streams and happy valleys; 'babbling of green fields,' as if his spirit had been already recreating itself in the gardens of Elysium. It not unfrequently happens that the spectra owe their origin to contemplations of future existence; and, consequently, that the good man's last hours are cheered with beatific visions and communion with heavenly visitors.

'Saw ye not even now a blessed troop

Invite me to a banquet, whose bright faces

Cast thousand beams upon me, like the sun?

They promised me eternal happiness,

And brought me garlands, Griffith, which I feel

I am not worthy yet to bear: I shall assuredly.'

*King Henry* VIII. *Act* iv. *Sc.* 2."

"One of the most curious instances of derangement that we have met with occurred in a phthisical patient. It consisted in a morbid association of ideas by mere similarity of verbal sound, or in other words a propensity to rhyme. Every person who came to the bedside was sure to receive a distich in honor of his name; nor could any remark be made in his presence without his seizing one of the words uttered and finding a rhyme for it, in doing which he exhibited great ingenuity. We were unable to ascertain whether he had been addicted when in health, to attempts at metre. Recitations of poetry, appearing to recur from a passive process of memory, with perfect unconsciousness of what is passing around, are frequent occurrences; and the passages selected have often a singular coincidence with events in the life of the moribund rehearser. Sir W. Scott's touching picture of the death of Madge Wildfire has had many unfictitious counterparts.

Dementia or imbecility sometimes comes on a short time before death. It is for the most part manifested by an incapacity of concentrating the ideas upon any one subject, and by an all but total failure of memory. The study of the degree of this condition necessary for invalidating a legal document is of great importance to the medical jurist. The mental weakness is in no respect so painfully exhibited as in the facility with which the subject of it derives pleasure from puerile amusements. 'Playing with flowers' is a token of approaching dissolution enumerated by a dramatic author, one whose observation pervaded human nature in all its phases. We remember visiting a lady in the last stage of a urinary disorder, during the progress of which she had evinced both strength of mind and refinement of taste:—We found her arranging with great care, and with demonstrations of delight at her success, a garland of flowers around a chamber utensil. A more humiliating spectacle could scarcely be witnessed. We augured that her decease was near at hand, and she died on the following day."

There are several phenomena displayed by the dying, which have always attracted the attention even of ordinary observers. The typhoid patient is observed to catch at minute objects in the air. There can be little doubt that the spectra are either the result of the state of the circulation in the eye or optic nerve, or of the inspissation of secretions on the cornea. Whatever the cause in any particular instance may be, in the great majority, it is certainly physical.

Picking at the bed-clothes, or, as Dame Quickly calls it, "fumbling with the sheets," is another notorious sign of coming dissolution. Dr. Symonds supposes that it depends on the revival of tactual sensations. We have often watched the dying patient engaged in this operation. In many instances it has seemed to us, that it was the vision which was false, rather than the tactual sense. We have seen the eye fixed on a portion of the coverlid, and gazing vacantly, but intently, on it, the fingers have begun to fumble and to pick it. In other instances, the act has not corresponded with any apparent operation of sight, but, whilst the eyes were closed, or fixed upon the ceiling, the hand and fingers have been at work upon the bed-clothes. In such cases, the explanation of Dr. Symonds is probably the true one. A little reflection will prevent surprise at the exhibition of phenomena of this sort. When we contemplate the irregular states of circulation, and the influence which its irregularity always exerts on the nerves of sensation—and, when we couple with this the disturbed operations of the mind itself, independently of present objects of sense—when we allow, in short, for the operation of delirium and of physical conditions, we shall feel little difficulty in accounting satisfactorily for what we see.

It is a question in which we all experience a melancholy interest, whether the act of dying is painful. Reasoning, facts, all forbid us to suppose that the mere act is so. But the agony preceding death is, in many cases, not only shocking to the stander-by, but, if the expression of the countenance and the ordinary gestures of instinct and of passions be not altogether false, it is one attended with much suffering. No doubt, all depends upon the mode of death—upon the functions first impaired—upon the nature and duration of the syncope. It must, we think, have been generally noticed, that women die more tranquilly than men. The reason, perhaps, is not hard to guess. The physical struggle is not so severe, and the minds of the fairer and more amiable sex are less disturbed by religious doubts, and by reflections on former vices or crimes.

We need not follow Dr. Symonds through his enumeration of other and familiar signs of death. The muscular debility; the weakened voice—the rapid, feeble, or irregular pulse—the difficult respiration—the loss of animal heat—the altered or arrested secretions—the changed countenance, are all well known, and more or less easy to be understood. Yet there is one point on which we may pause.

"The moribund are often impatient of any kind of covering. They throw off the bed-clothes, and lie with the chest bare, the arms abroad, and the neck as much exposed as possible. These actions we believe to be prompted by instinct, in order that neither covering nor even contact with the rest of the body may prevent the operation of the air upon the skin. There are actions and re-actions between the air and the blood in the skin, similar to those which occur in the lungs, and hence, in asphyxial disorders, the symptoms alluded to are very marked; but the mere influence of the air upon the cutaneous nerves has been proved by Dr. Edwards to be beneficial to the vital powers. Certain it is, that these symptoms are sometimes prominent in cases where the respiration is very little involved in the mortal struggle. Orfila, in one of his cases of poisoning by sulphuric acid, mentions that the subject of it made constant efforts to remove even the lightest kind of covering."

We fear that it is problematical, whether the aëration of the blood upon the surface has any thing to do with this phenomenon. Dr. Symonds proves too much, when he shews that it is present in cases where the respiration is very little involved. What the skin does is as nothing, compared with what the lungs do; and, if the latter perform their functions pretty well, it is the most improbable thing imaginable, that Nature should struggle for the fraction of a fraction of assistance from the skin. It is much more likely that the tossing of the arms and disposition to throw off all clothing, depend on a certain inexpressible anxiety felt at the præcordia or the chest. In no disease is this so striking as in the epidemic cholera. Whilst the skin is blue, and clammy and cold, the patient struggles against the least clothing on the chest, or even on the limbs. We asked a patient in this state, what it was which induced him to expose himself. He answered only by putting his hand on the lower portion of the sternum.

The signs of actual death form the concluding portion of the article. Some of *our* readers may recollect a notice of a book of horrors written by a clergyman, Dr. Whiting, and quaintly intituled—"On the Disorder of Death." In that book Dr. Whiting has painfully collected all the most celebrated instances of vivi-sepulture, and conjured up such frighful images of live-burials, as make us afraid of *dying* for fear we should be *alive*. In this country, where the bodies of the dead are preserved so many days before interment, reason must admit that the chances of being "nailed down" in our narrow cell, before the spirit has quitted for ever its tenement of clay, are slight indeed. Yet the curious diligence of Bruhier has collected fifty-two cases of persons buried alive, four of persons dissected prematurely, fifty-three of persons who recovered without assistance after they were laid in their coffins, and seventy-two falsely reported dead.

The indications of death are arranged by Dr. Symonds under three distinct heads:—

1st. Signs of the extinction of vital functions and properties.

2dly. Changes in the tissues.

3dly. Changes in the external appearance of the body.

1. The cessation of respiration and of circulation is both theoretically and practically unable to afford us distinctive evidence. For we cannot appreciate their minimum compatible with mere existence. Few, perhaps, would choose to apply M. Foubert's test, to wit, that of making an incision in one of the intercostal spaces, and feeling the heart with the finger!

The loss of animal heat is obviously uncertain too. But the loss of contractility of tissue is conclusive. Galvanism, observes our author, affords a certain and ready method of detecting this property. According to the researches of Nysten* irritability is first extinguished in the left ventricle; after forty-five minutes it has left the intestines and stomach; a little later the bladder; after an hour the right ventricle; after an hour and a half the œsophagus; after an hour and three quarters the iris. It next takes leave of the muscles of the trunk, then the lower and upper extremities, and lastly the right auricle. The duration of contractility is shortened by a warm and humid state of the atmosphere, by ammoniacal gas, carbonic acid, and sulphuretted hydrogen. It is unaffected by carburetted hydrogen, chlorine, and sulphurous acid; nor is it found diminished in cases of asphyxia by strangulation and immersion. The annihilation of that particular kind of contractility of tissue, which is equally distinct from muscular contractility, irritability, and elasticity, is one of the surest signs of death. We see it wanting in the collapsed edges of a wound which has been inflicted on the skin of a dead body, as contrasted with the gaping appearance of a similar lesion made during life.

2. The first alterations in the physical properties of the solids after death are softness and flexibility, to which succeed sooner or later the opposite conditions of firmness and rigidity. The cause of the rigidity of muscles is uncertain and has occasioned much dispute. Some regard it as the last effort of vitality. Others look on it as analogous to coagulation of the blood, if not the result of its coagulation. Yet that coagulation has itself been deemed vital, or the active cessation of vitality. The fact is, that we know very little of the matter, and we therefore pass on. This rigidity is pretty certain evidence of death—the flaccidity that succeeds to it is quite so.

Putrefaction, genuine putrefaction—is an unequivocal sign of death. It must not be confounded, though it has been so, with sphacelus.

3. The state of the surface is the last to receive the attention of our author. There is nothing in his observations to arrest us. Yes, there is a short summary of the appearances displayed at different periods, which may lead us to guess with an approximation to accuracy, the period that has elapsed since dissolution. The summary in question is M. Devergie's.

"We may suspect that the body has been dead from two to twenty hours if there be flexibility, elasticity, heat, and contractility; from ten hours to three days, if there be rigidity of the joints,

* Recherches de Physiologie et de Chimie Pathologique.

pitting of the soft parts, the natural colour of the skin, loss of animal heat, and no contraction under electric stimulus; from three to eight days, if there be flexibility (after rigidity) and no contractility; from five to twelve days, if the soft parts are puffed, elastic, and shining. After the twelfth day there is usually a separation of the epidermis, as well as a green tint of the abdominal integuments. But no certainty must be attached to these statements; they are merely approximative. The modifying influence of external media upon putrefaction is all but unbounded. In summer as much alteration may take place in five or six hours, as in eight or even fifteen days of winter."

We take our leave of Dr. Symonds. Though our parting is in death, it is not a very gloomy one, for the Doctor shews by the freshness of his intellects, that the signs of approaching dissolution are not to be looked for on him. Long may they be absent. The only fault we find with him is a crabbedness of style, which has latterly crept into our scientific writings. They have become a compound of French and English, wanting the sprightliness and vivacity of the one, and the manly terseness of the other. Our Encyclopædists write in an emasculated idiom, which reminds us strongly of the style of the Transatlantic Journalists. If we take up a number of the American Journal of the Medical Sciences, we are struck with the similarity. The reason is clear and the same in both cases. The study of French Medical Literature has generated this mongrel mode of writing. The French is the language of conversation. Even Madame de Staël scouted it as one of science. What then must this Anglo-Gallic tertium quid be? It came in with that pre-eminently dull book, the Cyclopædia of Practical Medicine. We wish we saw any probability of its going out again.

---

### CEREBRO-SPINAL IRRITATION, CAUSING MENTAL DERANGEMENT, TRISMUS, AND PALSY.

An interesting case of this kind is reported by Mr. A. Cockburn, in our respected contemporary of the North.

Christina Ferguson, aged 25, had been labouring under an affection of the spine for nearly two years, being confined to bed, with little or no power of the lower extremities. Fourteen days previously to Mr. T.'s attendance (27th June, 1835), she was seized with convulsive movements of the muscles of the neck and lower jaw, ending in trismus. Issues had been applied to the spine, and various medicines had been taken internally, without benefit. She had been a long time hysterical, with constipated bowels, and irregular catamenia. She was pale, languid, with dull eyes, dilated pupils, pulse 90. Castor oil was given to open the bowels. On the 28th he did not see her. On the 29th he was surprised to find her out of bed, walking about the room, but quite maniacal. She tried to get out of the window, and became violent when coerced. She was confined to bed—her head shaved—cold lotions and blisters applied—brisk purgatives administered. 30th, still very violent—trismus continues—had antimony in full doses. There was very little abatement of the maniacal violence till the 12th of July when, being much exhausted, she fell into a sound sleep, and awoke free from mental derangement, and also from the recollection of any thing that passed during the preceding fortnight. On the 13th she again lost the use of her limbs. This state continued till the 27th, when she became again deranged, with restoration of power in the lower extremities. On the 4th of August she became quite collected. She could move her limbs in bed, but could not stand. On the 8th she could walk a little. On the 18th she was convalescent. She went into the country for some time, and returned quite well. The treatment consisted chiefly of extensive blisters to the head and spine, with strong purgation. Menstruation, which had stopped, was restored.

Dr. Craigie, who had seen this patient, has appended a long and erudite commentary on the case, which, though curious and interesting, is by no means singular. We apprehend that all we know of its nature or pathology may be included in a very small compass. Dr. C. thinks the phenomena, in the early part of the disease, at least, "proceeded from irritation of the spinal chord, or its envelopes, or, perhaps, to speak more to the fact, from a languid and inert state of the circulation within the rachidian vessels, and a consequent degree of congestion, which first irritated and then compressed slightly the spinal chord and the origins of the nerves." The whole of the phenomena, during the paralysis of the lower extremities, and afterwards when mania was present, he attributes to irritation rather than to inflammation. Although we are inclined to take the same view of the case as Dr. Cragie has done, we are by no means sure that either he or we are quite authorised to deny the existence of inflammation as an element of the pathological condition. The shifting of its seat from the spine to the brain, and *vice versa*, is no proof of pure and sole irritation. We think inflammation, as erysipelas, exhibit the same vagrant propensities. And, after all, what is irritation but a degree—perhaps the first stage of inflammation? This is all but admitted by Dr. Craigie himself, in his definition of irritation. It is, says he, "a state induced by stimuli or irritants, in which the natural properties are unduly and unnaturally roused—yet, without giving rise to the process of inflammation, *or the formation of morbid products*." Now we apprehend that inflammation may, and does actually exist, in every case, before the "formation of morbid products"—and is very often quelled before these *consequences* ensue. A part is red, hot, painful, and swelled—exhibiting the real and essential characters of inflammation—and yet no "morbid products" are formed. And if this redness, heat, swelling, and pain be removed, without suppuration or effusion, are we to deny the existence of inflammation, and insist on the whole being *irritation?* We think that, in the case first detailed, it would be hazardous to aver that none of the elements of inflammation entered into the pathological condition.

---

### Inversion of the Uterus.

The following interesting case has been transmitted to us by Mr. Bloxam, an active and a very intelligent surgeon at Newport, in the Isle of Wight. We feel great pleasure in inserting it.

*To the Editors of the Medico-Chirurgical Review.*

Gentlemen,—If a space can be afforded in your valuable pages for the accompanying case of inversion of the uterus, it may perhaps contribute somewhat towards a more thorough comprehension of this formidable complaint; the diagnosis, treatment, and prognosis of which, are yet involved in some degree of obscurity.

I am, Gentlemen, your obedient servant,

JOHN E. BLOXAM.

*Newport, I. W. Oct.* 3, 1836.

In July, 1835, my father and myself examined the uterus of a woman, whose health and strength were very much reduced by hæmorrhage from the vagina; this hæmorrhage had recurred, at irregular and uncertain intervals, for several months, being sometimes very profuse, and at others in moderate quantity, with a great deal of mucous discharge during the intervals. We found a tumor, of a rounded but elongated form, protruding from the os uteri about an inch and a half; it was decidedly larger in circumference towards its lower extremity than where it passed through the os uteri—its upper part or neck continuing to diminish in size as far as the finger could follow it; it bore handling and even pinching without pain; pressure with the nail occasioned some pain, but it was by no means so sensible as the os uteri; it was covered with a smooth, slippery, tense membrane; and was easily moved from side to side, but appeared to possess such a degree of elasticity as to sustain its own weight, without resting on the adjacent parts; the os uteri was soft, and allowed the finger to pass between it and the tumor, but not so far as to enable us to ascertain what sort of attachment it had to the uterus. We formed the opinion that the tumor was a polypus, and I believe that all the published accounts of the diagnostic symptoms of this disease, would justify the opinion. We decided upon removing it with the ligature, which was accordingly applied, by means of the canula, in the usual manner, and drawn quite tight. The operation appeared to give the patient but little inconvenience, and we left her a few minutes after; but in little more than an hour, we were informed that she was in a great deal of pain; we went to her immediately, and found her expressing, both by words and in manner, the most intense suffering—complaining of great pain both in the back and hypogastrium; indeed, although naturally patient and composed in her manner, the energy and wildness of her expressions gave hei the appearance of a maniac: we removed the ligature, and she immediately resumed her usual composure, and declared herself to be in heaven. There was no return of pain, nor did any other unpleasant symptom appear.

We now made some further enquiries into the history of the case, and finding that the hæmorrhage had been recurring ever since her confinement, in January preceding; that she was then attended by a midwife; and that a profuse flooding took place soon after her delivery, followed by alarming faintness, which continued for many hours; we considered that, although these circumstances were all very compatible with the disease being a polypus, they confirmed the suspicion which the symptoms produced by tying the ligature had given rise to, viz, that the tumor was in fact an inverted uterus: we therefore examined the part in a different manner to what we had done before, and one which would probably always suffice for the diagnosis of these two complaints; we passed a ligature loosely round the tumor, in the same manner as we had done before, and then drew down the tumor by means of the ligature; the effect of this was, entirely to obliterate the passage which had existed between the os uteri and the tumor; the uterus being hereby completely inverted: the vagina, at the same time, was reflected upon itself, at its upper part, forming a cul-de-sac; and by passing the ends of the fingers into this, the absence of the uterus in its proper situation was satisfactorily ascertained. Either of these phenomena was, of course, sufficient to decide the nature of the case; and they were detected, in this instance, without either pain or difficulty.

The patient was a young married woman, the mother of several children; her health and strength were continually sinking under the effects of the injury; she was willing to submit to any thing, for the sake of her children, that was likely to prolong her life; we therefore determined, after explaining the risk and consequences to the husband, to remove the inverted portion of the uterus—which was nearly the whole of it—by means of a ligature.

Aug. 5th, 8, a. m., about a fortnight after the previous operation, we applied a large-sized cat-gut, (the base-cord of a harp,) round the narrowest part of the inverted organ, by means of the same sort of instrument we used before; the ligature was not drawn so tight as on the former occasion; it was only tight enough to occasion moderate pressure, which was done without much pain: in less than an hour the pain became severe, but by no means so bad as on the former occasion; an anodyne was given her, (morphiæ muriatis gr. ½,) after which she became easier. In the evening there was increase of pain, heat of skin, and excitement of the pulse, which ranged from 95 to 100 throughout the day: the anodyne was repeated, and she remained easy till midnight.

6th. Obtained no sleep last night; a good deal of pain came on at midnight, which lasted for some hours; after this she remained tolerably easy, during the whole of the day; vomited several times. Morph. mur. gr. j. in the evening.

7th. Slept comfortably during the night; pain very trifling; looks cheerful; pulse from 100 to 105, with more fulness; skin soft, moist, and cool; tongue clean, nausea continues.

8th. Pain inconsiderable; appears cheerful; symptoms all favorable. The ligature tightened, by drawing down both its ends, and tying a piece of small-sized whip-cord round them, each end having a knot at its extremity, to prevent the whip-cord from slipping off; each end being thus shortened as much as the diameter of the whip-cord. After five hours of ease, the pain recurred with severity, extending down the thighs, and continued for three or four hours. Pulse not quite so frequent or so full in the evening as on the 7th. Morph. mur. gr. ½ at bed-time.

9th. Obtained three or four hours good sleep; no nausea; appetite improved; other symptoms the same.—Ligature shortened, in the same manner as yesterday. In the evening complained of a good deal of pain in the back, hypogastrium, and down the thighs. Morph. mur. gr. ½ at night.

10th. Had very little sleep, and suffered a good deal of pain during the night; complains this morning of pain in hypogastrium and thighs; pulse 120; in other respects doing well. Ligature shortened as before; after this the pulse regained its usual rapidity, (from 95 to 100);—appetite improving. Morph. mur. gr. j.

15th. Sleeps well, and has been almost free from pain since last report; symptoms all favorable. Ligature shortened in the same manner daily, but considerable force was required to render it any shorter to-day. Feels herself much improved in strength since the operation was commenced.

18th. Going on well. To-day, for the first time, we were unable to shorten the ligature.

19th. Ligature shortened again to-day.

21st. This evening the instrument came away, with the tumor attached to it in the form of a mere shred; the anatomical structure of which, however, could be made out without difficulty; the cavity of the uterus, the fallopian tubes, the mucous and serous membranes, could all be easily traced. On passing the finger per vaginam, no orifice, corresponding to the os uteri, could be found; a hardish body was discovered in the situation of the uterus, but which, at this time, retained none of the anatomical character of this part. On the 26th, however, the orifice and the two lips of the os uteri could be distinguished, but the os uteri would not admit the finger.

Sept. 27th, 1836. Her health and strength, which underwent considerable melioration during the first few weeks after the operation, have continued gradually to improve up to the present time. She has not been subject to any periodical pain or indisposition, nor has there been any discharge resembling the catamenia till about a fortnight ago, when a coloured discharge made its appearance, and continued to flow for a few days; it was small in quantity, and of a more florid colour than usual: this occurred a day after she had been a good deal agitated and alarmed by some information that had been communicated to her. She is subject to giddiness, nausea, and some hysterical symptoms, which she attributes to flatulence, has frequently slight pains shooting down the legs, and a pricking darting sensation in the breasts and in the genitals, which extends from the latter down the legs, and is sometimes so violent that she can scarcely refrain from crying out, though the sensation is not that of pain. She is more subject to lowness of spirits, more easily agitated and alarmed, suffers more from any thing that is calculated to frighten her than was formerly the case. She at one time suffered very severe pain in the loins for several hours, in consequence, as she states, of having been told that a house in the neighbourhood was on fire. She says she has suffered less from these affections, since the appearance of the coloured discharge. There is a sense of weakness and bearing-down, if the bowels become confined, if she attempts to lift any heavy weight, if she exerts herself in any manner long together in the erect posture, or if she places herself in a kneeling posture; these symptoms of local debility have, however, much diminished during the last two months. A daily, though not constant, discharge of mucus still continues in very small quantity. She has sexual intercourse with her husband, but it appears to be in some respect in an imperfect manner; she says, she does not *think* it can be possible for her to become pregnant. About six weeks ago, I had an opportunity of ascertaining the anatomical state of the parts, and found that the upper portion of the vagina, with the remains of the uterus, had descended considerably nearer to the os externum; the os uteri was very distinct, its lips were become very thin, and projecting considerably into the cavity of the vagina; the top of the finger was admitted into the hollow formed by the lips, beyond which there seemed to be firm and perfect union, but the strength of the union was, of course, not put to the test of much violence.

The details of this case appear to me to afford sufficient evidence, that the most eligible method of removing so large a portion of the uterus by ligature, forms an exception to a valuable rule. In removing parts of the living body in this way, it is generally important, to apply the ligature at once, as tight as possible; by so doing, the vitality of the part is at once destroyed; or, at least, sensation and all the functions of a living part are entirely suspended, and absolute death quickly follows. But the uterus is an organ, over which the nervous system exercises an especial influence; it is subjected, at times, to great mechanical pressure; and, either it is able to retain its sensibility and vitality under such a degree of constriction as would destroy these properties in other parts, or else we have no adequately powerful means of applying a ligature to the uterus; or perhaps there may be a combination of these two conditions. The functions, of the whole of the nervous system, are very much affected by the influence of mechanical pressure; and the effect produced by any alteration in the amount of pressure, is greater, the more suddenly the change takes place: although one degree of pressure shall produce symptoms comparatively trifling, and, at the same time abate, suspend, or entirely destroy many or all of the vital functions of the part concerned; yet a slighter degree of pressure will produce symptoms of the most violent and alarming character, and without obstructing the usual functions of the part: the difference in the two cases being that of annihilation, or great excitement of the nervous influence.

If the utmost pressure that a ligature applied to the uterus can accomplish, fall short of that which is requisite for cutting off effectually the communication which is carried on through the medium of the nerves, the proper practice then will be, to obtain that degree of pressure which is required, in the most gradual manner possible. It might be anticipated that such a method of operating, although productive of less pain, would be attended with greater danger, in consequence of its greater tendency to occasion inflammation; but the above case sets this point as well as the former one at rest, as perfectly as an individual case can do: there was some nervous disturbance and excitement manifested during the operation, but there was not at any time the appearance of any considerable inflammatory action; moreover, the uterus itself after its removal did not exhibit the slightest appearance of inflammation; the peritoneum, even where immediately pressed upon by the ligature, had not united; this membrane was not merely discernible, but it appeared quite uninjured, with its ragged edge, where it had separated just above the ligature, distinct; it was puckered into folds by the pressure of the ligature, and even these folds seemed to require delicate handling to prevent their obliteration.

The operation that I have detailed does not appear to be under any disadvantage, even as regards the length of time required for its completion; the whole process occupied 16 days and 12 hours, which is a shorter period, I believe, than is usual in the ordinary method of operating.

If I should ever perform the operation again, I would adopt some further precaution in order to lessen the constriction at the first application of the ligature; perhaps soaking the cat-gut in water previous to using it, would be the only precaution requisite. The material that we used has many advantages over any other, but it has these two defects; first, it is of too unyielding a natuie; and secondly, it must be subject to some contraction after its application owing to the moisture of the parts; its want of suppleness prevents it from conforming to the shape of the part round which it is coiled, makes it more troublesome to apply, or occasions some degree of uncertainty in the degree of tension the loop may be in: by soaking the base cord of a harp in water, or putting it on the stretch by means of a suspended weight, for the purpose of rendering it straight or smooth, a ligature would be obtained combining every desirable property for this operation.

---

### Spectral Illusions.

A gentleman in the law, aged about 34 years, and accustomed to live rather freely—namely, to drink a bottle of wine daily—sometimes more—fell and struck his head against a wall. He was slightly stunned, for a few minutes, but resumed his avocations. He was advised, however, to take some aperient medicine, and to leave off his wine. This he did. In a few days he began to be sleepless at night—this vigilance increased, and soon afterwards, while lying awake at night, he began to see figures moving about in his chamber. They increased in number, conversed, laughed, argued, and harangued upon all kinds of subjects, and were often highly entertaining. He generally took notes of the conversations, and some of them were very curious. One night there was a large company of choice spirits, and the subject was punning. They kept it up for some hours, and he wrote down several of the puns, some of which were by no means despicable. In a night or two after this, the scene changed, and instead of being amusing, the Dramatis Personæ became most disgusting and even horrible in their words and actions. The gentleman now took alarm, and applied to Dr. Johnson. He affirmed that these illusions were not dreams. He was wide awake all the time, and often noticed the conversations of the actors to his wife who slept in the same room. If he or his wife got up and opened the window-shutters, the illusions vanished.

On examination, this gentleman's head was found to be hot, the eyes red, the tongue furred, the pulse rather quick—in fact, he was feverish. It was also observed that his hands trembled, like those of a person labouring under delirium tremens. He was ordered to be cupped in the nape of the neck—to take saline aperients—mild diet—and to drink three glasses of sherry daily. It appeared to Dr. Johnson that this gentleman was affected by a kind of delirium tremens, caused by the sudden change from a bottle of wine daily, to water. It is also probable that some injury was sustained in the head by the blow. Opiates were not directed till the febrile symptoms subsided.

He soon recovered on the above plan, and has ceased to consult Dr. Johnson.

---

### Case of Spectral Illusions. By Mr. Craig.

Since the foregoing case was written, the following very curious statement has been published in the Edinburgh Journal for Oct. 1, 1836.

A gentleman of great mental endowments, and master of 14 languages, then in his 76th year, began (1819) to have daily visitations of spectral images—at first occasional, afterwards more constant. In general, they presented human countenances and figures—the head and upper parts being much more defined than the lower. He rarely recognized any of his acquaintances.—The dresses were various, but generally antique, and consequently such as he had seldom seen, except in paintings. His own countenance was sometimes presented to him, gradually undergoing all the changes from youth to senectude. The figures were seen at different times in the day, and equally well when the eyes were open or shut. They were almost always of an agreeable character, and afforded a source of amusement. He had the power of calling them up, and of banishing them at pleasure. They were sometimes as large as life—sometimes much smaller. From the commencement of the spectral images, the gentleman continued his usual practice of taking little or no wine—if he transgressed, the number of images increased, and also their vivacity. They continued to recur for a series of twelve years, with very little change of character. His mental faculties appeared to be very little deteriorated—not more at least, than might be expected from age. When turned of eighty, he journeyed to London, "to dine with the Knights of the Bath," and returned by post at the rate of one hundred miles a day! He had now, when nearly ninety, great anxiety about a pension, the diminution of which was menaced!!!—His memory became very much impaired; but recovered to a considerable extent. When the cholera broke out in 1832, he was President of a Board of Health—and rigidly adopted the stupid rules of Cholera Boards in general. On the 21st August, 1832, while sitting at dinner, he lost the power of speech, but without loss of any sensorial power. A long detail of symptoms follows, which are little interesting. He was visited at various times by Mr. Liston, Dr. Abercrombie, Dr. M'Intosh, and others, who prescribed blisters, aperients and other remedies that seemed very proper.—When a little power of utterance was acquired, the language was a jargon of the 14 languages, which nobody could understand. Afterwards, however, he became more intelligible, and informed his attendants that the spectral images continued. One night he saw his wife traversing the room, and beckoning him to follow her. She had been dead many years. She seemed to glide out of the window, and through the same aperture he jumped out, and fell on the grass, from a height of seven feet and upwards. He got up immediately, and pursued the spectre through the conservatory and other places which had been her usual resorts. On being reminded that his wife was dead, he suddenly recollected himself, and mentioned the circumstance no more. He amended considerably, though he could seldom make himself understood as to his wants or wishes. His symptoms were invariably aggravated by disordered digestion or bowels. A question arose as to the sane state of his mind, and whether or not he was capable of executing deeds, &c. It was determined that, though he was unable to sign his own name, or give any intelligible directions, yet that he was of sane intellect. Mr. Craig's opinion was, that "his thinking faculty was quite correct, but the power of expressing it wrong." We believe that this definition of the nonagenarian's state was as near the truth as possible. His condition was nearly the reverse of the maniac or monomaniac, whose notions are wrong, but who is very capable of correctly expressing those notions.—From March till May, 1834, he went on rather improving. In the Winter of 1835, his pulse became much more irregular than usual, and dropsical symptoms appeared, with some difficulty of breathing. In December of the same year, he fell down stairs, and was found in a state of insensibility, but recovered sense, apparently from hæmorrhage, from a branch of the occipital artery divided by the fall. In March, 1836, he became affected with symptoms exactly resembling *delirium tremens*. He walked about for 36 hours incessantly, imagining that people were in the room and under the carpet. He took a large dose of muriate of morphia, slept for eight hours, and awoke in his ordinary state. In July, 1836, he caught cold, followed by considerable fever, which lasted nine days, and ended fatally, on the 15th July, in the 93d year of his age.

"The body was examined on the 16th of July, in presence of Dr. Abercrombie, Mr. Craig, Mr. Flockhart, and Mr. Comb.

In opening the head, unusually strong adhesions were found between the *dura mater* and inner surface of the skull, which was removed with much difficulty. The portion of skull-cap which covered the right hemisphere was of moderate thickness. Considerable effusion over the surface and between the convolutions of the brain, and great vascularity over the whole, indicating extensive chronic inflammatory action, especially in the course of the superior longitudinal sinus, where the *dura mater* was uncommonly thick.

The brain was examined by cutting thin slices from the surface downwards, commencing with the right hemisphere, which was found sound throughout, with the exception of a small tubercle, about the size of a split-pea near the surface, and similar to what is often seen in strumous children. The right ventricle was also sound. The left hemisphere was examined in a similar manner, and in the posterior lobe of the brain there was a cavity rather exceeding two inches in length, running obliquely outside of the left lateral ventricle, lined with a yellowish membrane, and the brain around its circumference was much softened. The cavity was close to, but not communicating with, the ventricle, which was sound.

The remaining part of the brain was then removed, and the base examined, which also over its whole extent bore strong marks of chronic inflammatory action. The internal carotids at the junction of the optic nerves were ossified. The base was then examined by cutting off slices. In the middle lobe, on the left side, a little behind the pituitary gland, and to the left of it, was a small diseased appearance, similar to what was in the posterior lobe, not exceeding a quarter of an inch in extent. No other diseased appearance except the increased vascularity was recognized.

The *cerebellum* was sound. On the posterior part of the *medulla oblongata* there was a small tubercle.

The thorax was opened. The right lung was found remarkably healthy; but the left lung adhering to the pleura. The substance of both lungs was quite sound. A good deal of fluid was effused both in the *pleura* and *pericardium*. The semilunar valves of the aorta were considerably ossified, and the *aorta ascendens* was enormously large, almost aneurismal, but sound in structure."

Dr. Craigie has appended a commentary on this case of no less than 14 pages. The commentary is learned, recherché, and, we think, judicious. We would only hint that it is rather prolix. He very properly remarks that "the appearances on dissection only disclose the ultimate effects of morbid action, while a thick veil is thrown over all the intermediate steps of the process." Alas! how applicable is this observation to the generality of diseases! Still, a study of this, and of every case, may lead to some practical conclusions, and advance, step by step, our imperfect science.

Dr. Craigie properly oberves that spectral illusions are sometimes owing to gastric irritation, especially in hypochondriacal patients—probably attended, at the time, with disordered circulation in the brain. In the case above related there can be little doubt that cerebral disturbance was the cause. The dissection proved this sufficiently; but had there been found no traces of *disease* in the encephalon, it would not have been a proof that no functional disorder had previously existed. The illustrations, however, which Dr. Craigie has appended, are important, and will well repay the labour of perusal.

---

### Wound and Successful Ligature of the Internal Jugular Vein.*

This case occurred to Dr. Morgan, Professor of Surgery in Geneva College, New York. He has related it at considerable length in our American contemporary.

*Case.* The patient was a convict, æt. 25, confined for life in the Auburn State Prison. On the 31st of December he endeavoured to hang himself.

* Amer. Jour. of Med. Sciences, Aug. 1836.

On the morning of the 1st of January, he cut his throat.

When Dr. M. was summoned to him, he found him lying upon a bed, exhibiting the most ghastly appearance; the bed on which he lay, and the floor about it were literally deluged in blood; and there was an incision about five inches in length, commencing near the sterno-clavicular articulation of the right side, following close upon the superior border of the sternum and of the clavicle of the left side for about three inches, continuing in its extent upwards and outwards. The wound had been plugged with pledgets of lint, before Dr. M.'s arrival. In removing the folds of cloth from the wound, a prodigious burst of blood followed, as if the carotid and jugular had both been severed. Pressure was immediately reapplied as well as possible over the bleeding surface, and in a manner that checked the flow of blood. The pulse, which was greatly reduced before from this tremendous gush of blood, sank to a flutter, and the patient swooned. Restoratives were twice necessary. The second time, while the patient was in the state of syncope, Dr. M. was enabled to tie the superior thyroid artery, which had been divided, and to ascertain that the internal jugular vein was nearly cut across. With a blunt aneurysmal needle, he passed a ligature from within outwards, about three-eighths of an inch above the wound, protecting the nerve and artery with his fingers, and aiding the passage of the needle through the sheath by a gentle motion of the intrument opposed to the nail of the index finger of his left hand. The needle readily passed, and the vessel was secured. But the patient coughed incessantly, and when he did so, the the blood retrograded in a full stream from the subclavian vein.

"The ligature which had been applied to the vein, was now handed over to my assistant, whilst I attempted to secure the lower portion of the vessel; this was a point of some delicacy. The cut having been made close upon the superior edge of the sternum and the sternal extremity of the left clavicle, further dissection became indispensable.

The vein being put upon the stretch by the ligature; with a delicate knife having a convex edge, I commenced my incisions upon the scapular side of the sheath, (making the index finger of my left hand the director,) and, by the most guarded dissection, succeeded in detaching the vein from its connexions for about half an inch below the superior edge of the sterno-clavicular extremity of clavicle: I then passed a silver-eyed probe, armed and bent to suit the situation, dawn upon the outside of the sheath, close upon the vein, until the end of the probe appeared opposed to the superior portion of the sheath between the artery and vein; by then scratching with my finger nail over the extremity of the probe, it readily passed. After being fully satisfied that nothing was included but the vein, I tied the ligature, and all hæmorrhage ceased at once.

In now examining the wound more particularly, I found the internal jugular vein divided in about three-fifths of its circumference, the external jugular and the superior thyroid artery completely divided; the sterno-cleido mastoid muscle completely, and the sterno-thyroid partially severed. The razor, in passing from right to left, laid the sheath bare, but did not sink sufficiently deep in its course to injure the coats of the artery, and yet left the free opening in the vein."

We need not particularise the method of dressing the wound, nor the subsequent diurnal reports. There is nothing of moment in one or the other. On the 6th day, the ligature separated from the superior thyroid artery. On the 7th the upper ligature came away from the vein; on the 9th, it was followed by the lower. On the 20th, he returned to tailoring, his usual employment: and we may trust that this convict will cut coats in future and not his throat.

"The cases recorded," says Dr. Morgan, "where the internal jugular vein has been successfully tied under any circumstances, are few in number. I know of but two instances in which this operation has been successfully performed in this country besides the case under consideration.

One of these was performed by Dr. Stevens of New York, in 1830; the other by Dr. Gibson of Philadelphia, in Nov. 1832. In the last case, the ligature was applied in consequence of the vein being involved in a tumour, 'occupying the whole of the left side of the neck,' in the removal of which it became necessary to detach the vein, or secure it with a ligature."

---

### New Kind of Truss for Prolapsus Uteri.*

Dr. Annan, of Baltimore, has published an account of a sort of truss that has answered very well in this unpleasant complaint. It is a modification of the Truss for Prolapsus Recti. We must therefore describe the latter first.

The upper part resembles the spring and main strap of a common double truss, wanting the pads, and is designed to embrace the sacrum and the wings of the ilia. Opposite the base of the lumbar vertebra, a curved spring is attached at right angles to the upper part, and after following the curve of the sacrum it terminates in a pad intended to act upon the anus. This rectal truss was modified thus by Dr. Annan.

"The truss, I have employed," we are at present speaking only of the rectum, "differs from the foregoing, in having a circular plate riveted to its centre, which has two narrow straps fastened upon it, under which the curved spring slides, and a small screw passing through holes, fastens it, so that by slipping it up or down the pressure upon the anus can be increased or diminished at pleasure. I also found it necessary to take the temper out of the curved spring, in order that the patient might bend it to any shape to suit the curvature of the sacrum. In consequence of this loss of elasticity, it had to be made somewhat stronger to make sufficient pressure."

Thus far he had modified the rectum truss, and thus he used it with much benefit in a case of polapsus recti. But we are to shew how he came to apply it to prolapsus uteri.

"Having at the same time a patient with the uterus in that state of descent which has been termed procidentia, on whom various pessaries had been tried, with temporary relief, but who, for a considerable period, had been unable to wear any of them, it occurred to me that the same instrument somewhat modified might answer for the support of the uterus. The first trial was made by lengthening the curved spring, so as to throw the pad forward in front of the anus upon the perineum. This gave some support, but was found to be extremely inconvenient in walking, and in sitting down was apt to pinch the nates, and she refused to wear it. It then struck me, that, by reversing the whole apparatus and turning the curved spring in front, it possibly might answer better. The curved spring was accordingly greatly reduced in length, and she was directed to wear it in front; but she now complained that it passed in between the labia and caused great irritation and profuse leucorrheal discharge, and, when she sat down, the pad struck the chair, and incommoded her exceedingly. The curved spring was still farther shortened, and the pad made to press upon the posterior commissure and anterior portion of the perineum, thus removing the obstacle to sitting down comfortably; and so great a degree of curvature was given to this spring, that it lay outside, in front of the labia, thus obviating the other objection, and now the relief afforded was complete. The prolapsed uterus was perfectly supported, all the distressing symptoms were entirely removed, and, for the first time during many years, my patient was enabled to walk with ease and satisfaction. She has now been wearing it about two years, and walks the greatest distances with an entire freedom from all the symptoms of prolapsus, under which she had previously endured worse than the pains of dissolution. She now wears it only when she goes out to walk; and before putting it on she wraps a clean linen or muslin cloth

* Ibid.

around it, which feels more pleasant than the oiled silk that covers the padding. When she returns, she takes it off, and does not find it necessary when going about the house."

"It remains only to say, that the curved spring should be eight and a half or nine inches long, from the lower extremity of the pad to the top of the circular plate upon which it slides; and it must not be forgotten that the tempering must be omitted, in order that the patient may give it a degree of curvature to suit herself, that it may lie outside of the labia externa."

Dr. Annan points out the advantages of this truss. We may leave their determination to actual trials with it, and content ourselves at present with simply introducing it to the attention of our readers.

---

### NEW MODE OF PERFORMING AMPUTATION OF THE PENIS.*

A negro, aged 27, had enlargement of the penis. It measured eight inches in length, and on its under side, from about three inches from the scrotum to the neck of the penis, the structure of the organ was changed so as to resemble cartilage, and so much enlarged as to throw the glans upwards until almost in contact with the dorsum of the penis, an inch above the neck. Upon the enlarged portion were two fistulous orifices, through which only a few drops of urine passed when the patient urinated, the greater portion of the water passing in a very small stream through the natural channel. The glans penis was of the natural size, and free from disease; its shape was somewhat altered from being turned upward by the pressure from beneath. The urethra was almost obliterated in the enlarged part from pressure, and there was a slight stricture at the bulb. Such was the state of the case when it came under the care of Dr. Ogier, of Charleston, South Carolina. Under his treatment it became worse, and he determined to amputate the organ. Yet he was anxious to preserve the glans, which it has been stated was sound. He, therefore, determined to cut through the penis above and below the diseased portion, to cut *it* out, and, applying the glans to the stump, to endeavour to unite the two. The "Nigger" was sufficiently anxious for appearances to seize the offer, and Dr. Ogier accordingly made the interesting attempt.

"The operation was performed on the 20th of June, 1835, in presence of Drs. Holbrook, Baron, Gourdin, Logan, and a number of students, in the following manner. I divided the penis transversely from below upwards about two lines above the neck—having divided the urethra, I cut carefully through the corpora cavernosa until only about one or two lines of the dorsal portion of this remained. I then turned the edge of the knife towards the pubis, and cut in a horizontal direction until I had extended the cut above the diseased portion, leaving a layer of the corpora cavernosa, about two lines thick and about four in breadth, attached to the skin on the back of the penis. The bistoury was then turned downwards, and the section of the penis thus removed. The glans was therefore left connected to the stump by a thin layer of the corpora cavernosa and the skin on the dorsum of the penis. The hæmorrhage was not very extensive, only two arteries requiring the ligature. The general oozing from the stump was easily arrested by dusting the parts with powdered resin. The wound was allowed to remain exposed to the air for five hours before the dressings were put on. The glans penis was then brought down and applied to the stump, and confined there by seven sutures. A silver catheter was introduced carefully so as to give support to the parts, as well as to prevent the urine from passing through the urethra; and, finally, the whole penis was enveloped in fine cotton, kept in place by a slight bandage.

I used the cotton more to keep the parts in contact than to preserve the heat in the glans; and in all wounds

---

* Ibid.

about the ear, lips, or nose, I have found it very effectual in keeping the parts in contact: the fibres of the cotton on each side of the cut become moistened from the oozing of the wound, and afterwards dry and adhere to the lips, and thus prevent all possible motion between them."

After forty-eight hours, the dressings were removed. The glans was doing well, except at the attachment of the frænum, where it was in a state of mortification. This slough came away on the fourth day, leaving the glans and urethra open on its under surface. By dint of a little trouble, the remainder healed well. Then the loop of skin and corpus cavernosum, on the dorsum of the penis, was tied with a tight ligature, and gradually sloughed off. Twice the mucous membrane of the urethra, about two inches from the end of the penis, became swollen and indurated, and it was necessary to apply leeches, and to keep a catheter in the urethra. But these symptoms subsided, and all went on well. In October, the negro went to his master's plantation in the country, and at the date of the report, May 1836, the disease had not returned. Before Sambo went back to the fields, he declared that the sensibility of the glans was by no means bad, and, being an inductive philosopher, he determined to put the point beyond dispute, by having connexion with a woman. We may presume that he was satisfied, for he made no complaint.

The operation is more a matter of curiosity, than one that is likely to be often practised or practicable. But a case like the present *may* occur, and the result of the present is worth knowing. Unfortunately, amputation of the penis, if necessary, is usually required for cancer of the organ. If that be cancerous, and the glans is unaffected, we should still be loth to leave it. The operation must be, pro tanto, less complete, and the chances of recurrence of the morbid growth must be increased.

---

### Modification of Dupuytren's Instrument for the Cure of Artificial Anus.*

Dr. J. R. Lotz, of Union County, in the United States, has proposed a modification of that instrument of M. Dupuytren's, with which most of our readers must be familiar. We need not relate the case in which it was applied, nor compare it with the late Baron's instrument. Its great advantage over the latter is said to be its much inferior weight. All that we shall introduce is the description of the instrument, and of its mode of application.

A figure gives a correct idea of the general form of the instrument. The two fenestræ† are about an inch in length, and a quarter of an inch in breadth, surrounded by a solid rim about a line in thickness. The whole instrument is about six inches in length, and from these data the other dimensions may be readily deduced.

They are articulated in the following manner. At the upper end (that which in forceps corresponds with the joint), and again in the middle of one of the blades, there are attached two steel slides which play loosely through mortice holes cut in the corresponding parts of the other blade. Near each of these slides is a screw; that at the lower end passes through one blade, and simply presses on the other, acting in such a manner as to regulate the distance of the blades from each other, while that in the centre of the instrument passes through both blades, approximates them, and causes the edges of the fenestræ to press against each other.

The mode of application is this. The central screw being removed, the blades are entirely detached. One of them is inserted into each of the intestinal extremities in this condition, and the slider being introduced into the mortice holes, the surgeon is assured that the fenestræ are equidistant from the orifice.

---

* Ibid.

† These are the blades introduced into the two portions of the bowel.

The central screw is now introduced, and the adjusting screw having been previously turned far enough to allow for the thickness of the double walls of intestine included between the pinching extremities, the central screw is tightened until the edges of the fenestræ press firmly upon the intervening membranes. By unscrewing the adjusting screw and tightening the central one, the pressure can be increased to any requisite degree without destroying the parallel direction of the blades.

We must own that we do not perceive what material advantage this instrument possesses over M. Dupuytren's. Both might be simplified. They are the same in principle. Surgeons have the opportunity of deciding for themselves.

---

Some Experiments and Observations on Tying the Carotid and Vertebral Arteries, and the Pneumo-Gastric, Phrenic, and Sympathetic Nerves. By Sir Astley Cooper, Bart.*

Our old friend Sir Astley is of far too active a mind to be doing nothing. He is still anxious to improve a profession that he has ornamented. The experiments to which we shall now direct attention are fresh examples of his zeal.

The powers of the anastomosing vessels are sufficiently established. Ligatures have been placed on all the great arteries with more or less success; the collateral branches being almost always fully adequate to carry on the circulation. Sir Astley's experiments are not therefore intended to prove that point, for it is proved. What they do shew will be shortly evident.

1. Experiments on the Carotid and Vertebral Arteries.

A. *Vertebral and Carotid Arteries of a Dog Tied—Animal killed nine months afterwards.*

On the 28th of January, 1831, Sir Astley tied the right and left vertebral and the right and left carotid arteries of a dog, and all was completed within half an hour. The animal appeared insensible, or as if it were intoxicated; it had difficulty in breathing; its pupils were dilated; its volition was diminished; and it ran against the leg of the table, or any other body, without seeing or regarding it. When placed upon its legs, it fell down on its right side, and had spasmodic twitchings of its hinder extremities.

At the expiration of a quarter of an hour, it was still insensible: it had shiverings, although placed near the fire: it rested its head upon the ground on the right side: its respiration was still laborious; and its pupils were dilated.

After an hour and a half, however, it was able to stand, and, although with difficulty, to stagger around a small room.

On the 29th it was dull, and indisposed to move. On the 30th, it was much the same, and not inclined to move or eat. On the 31st, it walked round the room; and ate about an ounce of food, but would not lap. On the 1st of February, it was much better: it ate and drank; and from that time gradually recovered. It afterwards became a good house-dog, and Sir Astley kept it for nine months, when he killed it and injected it.

The carotid artery on the right side was obliterated opposite the fifth and sixth cervical vertebræ: below the obliteration it is injected from the aorta; above the obliterated part it is filled with injection, (1) from the inferior thyroideal artery communicating with the superior thyroideal by large branches; (2) by a large descending cervical branch, dividing into numerous large anastomoses; and (3) by branches from the vertebral artery anastomosing with the external carotid artery on the first vertebra of the neck.

The left carotid was obliterated from near its origin, but filled with injection above the obliterated part, by the inferior thyroideal artery communicating with the superior, and by the ascending cervical artery from the subclavian, by

* Guy's Hospital Reports. No. III.

numerous and large anastomoses, and by an œsophageal artery from one of the intercostals communicating with the superior thyroideal artery.

The right vertebral artery was obliterated near its origin on the seventh cervical vertebra, but filled with injection above the obliterated part by two branches from the superior intercostal arteries, which passed, on the back of the spine, into the arterial canal of the vertebræ, at the fourth, fifth, and sixth intercostal spaces. The vertebral artery thus produced passed to the second vertebra of the neck, where it formed the basillary artery, and, in its course, had festoons or loops formed in it, as far as the first vertebra, at each intervertebral substance; and here, upon the transverse process of the first cervical vertebra, it formed communications with the carotid.

The left vertebral artery was obliterated close to its origin; but was filled with injection by an anastomosing branch from the superior intercostal artery, which entered between the fifth and sixth vertebræ of the neck; and by a second branch, also, from the intercostal artery passing on the posterior surface of the transverse processes of the fourth and fifth cervical vertebræ: then, over each transverse process was a loop of arterial communication, forming down each side a beautiful display of festoons.

The basillary artery began at the base of the second cervical vertebra; passed to the junction of the first vertebra to the head, where it again received vessels from the vertebral arteries; and then proceeded, as a single artery, to the points of the petrous portions of the temporal bones; where it formed the commencement of the circle of Willis, which was well filled with injection, and sent off its usual arteries to the brain.

The vertebral artery also joined the internal carotid artery on the transverse process of the second cervical vertebra of the neck.

We have been particular in recording the exact mode of anastomosis in this case, because it could hardly be anticipated, that so sudden and so violent a cutting off of the supply of the blood to the brain would allow time for the development of adequate anastomoses. Those anastomoses may be broadly said to be—the re-entrance of blood into the carotid, above the ligatures, by the communication of the thyroid arteries; and the re-establishment of the circulation in the vertebrals above the ligature, by means of the upper intercostal arteries.

On a second occasion, Sir Astley tied successively the two vertebral arteries, and, after an interval of eight days, the two carotids.

The animal was weakened in its fore-legs; but in other respects it suffered less than the former; and on the following days it took its food as usual.

The carotids were obliterated at the points where they had been secured, as were the vertebrals. But both were supplied above that spot by an ample collateral circulation which it is unnecessary for us to describe.

When the carotid *and* vertebral arteries were tied in the rabbit, that animal immediately died.

The next object of Sir Astley was, to ascertain the different effects that would be produced by tying separately the vertebrals and the carotids.

*Ligature placed on both Carotids.*

When the carotid artery on each side of the neck was tied, little effect was produced; except, that the respiration was quickened for a few minutes, and the animal rendered dull and disinclined to eat during the day: but on the following morning it appeared lively, and ran about with its natural activity.

Sir Astley conjectures that the quickening of the respiration may be attributed to a greater quantity of blood being impelled through the vertebral arteries, in consequence of its interruption in the carotids. May not the excitement of the operation, perhaps some slight disturbance to the nervus vagus in its performance, account for the circumstance in question?

B. *Ligature placed on both Vertebral Arteries.*

" I next placed a ligature around both vertebral arteries. When I had tied the first, there was some difficulty in breathing; but when I had tightened the second ligature, this difficulty was greatly increased. The respiration was at first slow, but it afterwards became quicker. The animal retained volition and sensation, but its fore legs were weakened.

At the end of two hours, its breathing was laborious; its ears dropped to the right side; its heart beat quickly; it was dull, and indisposed to move; and its fore legs were still weak.

After four hours and a half, it ran about, but with its ears fallen: its respiration was slower.

On the following day, there was a murmuring in its breathing, which was increased under excitement: its heart beat quickly and forcibly: its pupils were not dilated.

On the second day, the respiration was slow and heaving; it had an irregular action of the heart; it was dull; and its heat was 102. In the evening, its respiration was irregular and heaving; but it moved about, and took food.

On the third day it was dull: its breathing was slow: its heart beat quickly; it ate food.

On the fourth day, it appeared heavy, and indisposed to move. The action of its heart was quick and strong: its respiration was slow, but no longer stertorous.

On the fifth day, its breathing was slow: it appeared dull and heavy: the action of the heart was still quick.

On the sixth day, the respiration was laborious and slow, being only 64, instead of between 120 and 150, the natural number of inspirations, in a minute: its heart beat rapidly, and not forcibly: it was very dull, and indisposed to move: it was getting thin, but it took its food as usual.

On the seventh day the animal was found dead; and on the eighth I examined it, after injection, and found an abscess in the neck. The vertebrals had been well secured, and the brain had received injection by the carotid: the basillary and cerebellar arteries were filled from the circulus arteriosus. This animal's death may have been hastened by the abscess.

I have many times repeated this experiment; and it uniformly produces a marked effect upon the respiration, which it renders slow and laborious. The fore legs are weakened; and a much more severe effect is produced upon the animal than when the carotid arteries are obstructed; insomuch, that it will rarely recover from the operation."

C. *The Carotids first Tied, and then the Vertebrals.*

Sir Astley tied the carotid arteries. The respiration and circulation became quicker, and the former remained so for forty-eight hours, or a little more. After the fourth day it appeared quite healthy, and on the ninth Sir A. tied the vertebral arteries, which were obviously enlarged. The respiration stopped immediately, and the animal appeared dead; but it afterwards made several gasps, from convulsions of the diaphragm: its hinder extremities became also convulsed; but in a minute, all voluntary motion ceased.

On opening the abdomen and chest, it was seen that the peristaltic motion of the intestines remained; and the heart continued to act for a few minutes after apparent death.

D. *Carotids Tied—Vertebrals Compressed.*

Thinking that compression of the vertebral arteries might prove a substitute for the ligature of those arteries, which is difficult, Sir Astley tied the carotid arteries. Respiration was somewhat quickened, and the heart's action increased; but no other effect was produced. In five minutes, the vertebral arteries were compressed by the thumbs, the trachea being completely excluded. Respiration almost directly stopped: convulsive struggles succeeded; the animal lost its consciousness, and appeared dead. The pressure was removed; and it recovered, with a convulsive inspiration. It lay upon its side, making violent convulsive efforts; breathed laboriously; and its heart beat rapidly.

In two hours it had recovered; but its respiration was laborious.

The vertebrals were compressed a second time. Respiration stopped: then succeeded convulsive struggles, loss of motion, and apparent death.

When let loose, its natural functions returned, with a loud inspiration, and with breathing excessively laboured.

Compression was re-applied at the end of five hours, of nine hours, of twenty-four hours, and of forty-eight hours, with just the same effects as on the previous occasions.

E. *Vertebrals Tied—Carotids Compressed.*

After tying the vertebral arteries, the respiration became immediately laborious, the right ear fell, and the right fore-leg was partially paralysed. On the following day, the respiration was slow, and it appeared dull, but it had partially recovered from the other symptoms.

"I pressed, with my thumbs, the carotid arteries on each side of the larynx, which was left free.

It fell upon its side; it lost all sensation and volition; and its eyes were drawn back.

Upon removing the pressure, it soon recovered.

On the second day, its respiration was quick; its ear much risen; its fore leg less paralytic: it sat up; and moved from place to place.

A second time I compressed its carotids. Its eyes were drawn back; it was convulsed; and its respiration was quick and laborious; and it was affected in the same way as on the preceding day, but in a less degree.

On the third day, its respiration was hurried, and 150.

For the third time I compressed its carotids. It fell upon its side, and was insensible; but soon recovered, and ran about."

On the fourth day it was dull with laborious respiration. On the morning of the fifth it was found dead. Abscesses had formed around the ligatures. Coarse injection entered the cranium by the internal carotids, but not by the vertebrals; nor was there any injection in the basilar artery by the circle of Willis.

F. *Carotid and Vertebral Tied on the same side.*

"I tied the carotid and vertebral arteries on the same side; the breathing became laborious, and the fore-leg was partially paralyzed. I subsequently compressed the vessels on the other side, with the usual effect of producing apparent death: but the pressure being removed, the animal recovered; and at the expiration of eighteen days it was quite well, excepting that it had a difficulty in breathing, under excitement."

G. *Vertebral and Carotid Arteries tied at the same time.*

This was done. The animal breathed no more. There were only thirteen or fourteen convulsive contractions of the diaphragm, and convulsions of the hinder extremities.

"The same effect of interrupting the streams in the vertebrals and carotids was produced in an equally conspicuous manner, without the application of ligatures, as follows. The animal was held in a convenient position, with its neck extended, and its head thrown back. I then applied my thumbs, so as to compress, at the same time, the two vessels on each side, taking care to leave the trachea entirely free from compression. Respiration ceased in a few seconds: some struggling then took place, and the animal appeared dead. The pressure being then removed, the respiration was completely suspended; but artificial motion being given to the ribs, the animal gasped, began to breathe quickly, and recovered."

It might reasonably be asked, if pressure on the nerves had no effect in occasioning the preceding train of phenomena. Sir Astley properly anticipated this proper question, and proceeded to answer it in the proper way, by making experiments on those nerves themselves.

1. He placed a ligature on each pneumo-gastric nerve of a rabbit. The animal's breathing became heaving and laborious, and fell in eight hours from 130 to 48 inspirations in the minute: it was likewise accompanied by a stertorous noise: the heart beat feebly, but rapidly: food was refused. These symptoms continued; and on the following morning it was found dead. The same experiment was several times repeated, and the results were nearly uniform, the animals dying at the end of from nineteen to twenty hours.

In these experiments, it was likewise observed that the blood circulating in the arteries gradually assumed the venous colour, and that the animal heat at the same time decreased in a remarkable degree. At the time of the performance of the experiment the heat, in one rabbit was 104; at the end of 8 hours, it was 93¼, in the rectum; and at the end of 12 hours, when the rabbit died, it was 88½ in the abdomen. In a second rabbit, the animal heat at the time of the experiment, was 102; in 16 hours, it was 93. In three quarters of an hour more it died, when the temperature was 87 in the abdomen.

The examinations after death exhibited the lungs gorged with blood, and looking like the liver; a fluid in the pleural cavity; the stomach full of undigested food; and the œsophagus likewise distended with it, in those cases in which the animals had taken food after the ligatures had been applied.

Sir Astley divided the phrenic nerves in quick succession.

The diaphragm, of course, was paralysed, and the respiration was laboriously performed by the intercostal muscles, which heaved the ribs violently. In a quarter of an hour it lay upon its side, making great efforts with its intercostal muscles; and sometimes it stopped, as if fatigued, and then again commenced. In twenty minutes it was dead. The heart's action and the peristaltic motion of the bowels were observed for a short period after apparent death.

3. Sir Astley tied the sympathetic nerve in the neck.

The respiration became quick and irregular; but sensation and volition were unaffected. The heart's action was very quick: there was a general trembling; but, when the animal was put down upon the ground, it ate some greens. Its respiration continued irregular, and its heart's action very quick; and after eight days it was killed. The nerves were found well tied: one had ulcerated below the ligature; the other was nearly ulcerated through; and the ligature was surrounded by suppuration.

He tied these nerves in another rabbit, which, a month after the operation was still lively and active.

4. Sir Astley tied, in the same rabbit, the pneumo-gastric nerve, the grand-sympathetic, and the phrenic. The respiration became laborious; the animal dull, and indisposed to move; and the heart's action feeble. The breathing continued excessively laborious for a quarter of an hour, when the animal died. Bloody fluid was found in the chest: the lungs were not much changed. In another similar experiment, the animal died in three-quarters of an hour.

5. Sir Astley tied the jugular veins on each side of the neck in a rabbit. Its respiration was reduced to 68 inspirations in a minute, which is about half the natural number. After four hours, it ran about as if nothing had happened; and eventually recovered.

When killed and injected, three anastomosing veins passed from the anterior to the posterior part of the jugular vein, and conveyed the blood from the head to the heart. The vertebral vein too was enlarged.

In another rabbit, after the jugular veins were tied, the respiration became slow. For five days it seemed dull. On the seventh day, it was convulsed, rolled over frequently, lost in a great measure sensation and voluntary motion, and died. On examination, a clot of blood was found extravasated in the left ventricle of the brain.*

Such are the experiments performed

* Sir Astley has known this happen from enlargement of the glands in the neck of a boy.

by Sir Astley, with the view of determining the effects of arresting or diminishing the supply of arterial blood to the brain. The correlative experiments performed upon the nerves tend in a great degree to do away with the main objections which might plausibly be urged against those on the arteries. After making all reasonable allowance for the effects of the mere wounds, for disturbance of the system, and for violence committed on the nerves themselves, candour must admit that Sir Astley has succeeded in establishing positively, what before were comparatively suppositions—the important consequences of the ligature of the great cerebral arteries. These consequences may be thus summed up.

1. Tying both carotid arteries in the rabbit, does not seem to occasion any material effects. In the dog, the same operation is not followed by serious results.

2. When the vertebral arteries are tied in the rabbit, the respiration is rendered slow and laborious—the fore legs are weakened—the action of the heart accelerated—and the animal does not recover.

It is remarkable that very slight injuries, after a ligature has been put upon these arteries, will destroy life; insomuch, that if they are first tied, even dissecting for the carotids, without tying them, will cause death.

3. Compression of the carotid and vertebral arteries at the same moment, occasions in a few seconds cessation of the respiration. The same effect results still more decisively, if the four vessels are tied.

When the dog is the subject of this experiment, it loses its volition and sensation, and appears as if it were intoxicated; but the anastomosing vessels gradually restore the circulation, by means of the other branches of the subclavian artery at the back and sides of the neck.

Such are the results which appear to be very conclusively established by the experiments of the worthy Baronet.

It is not uninteresting to revert to the effects of tying the pneumo-gastric nerves. The diminished frequency of respiration—the loading of the lungs with dark blood—the venous character of that in the arteries, from which, when opened, it still flowed per saltum—and the fall of the animal heat, are all striking phenomena.* Are all to be explained by the primary influence upon the respiration, or is effect upon the heart, perhaps upon the blood itself, to be regarded as an important element of causation? Such questions are more easily asked than answered.

We cannot quit the subject without

* In an Appendix, Sir Astley has shewn that this diminution of animal heat is not constant. We extract the memoranda of three experiments.

" *Experiment* 4.

| | Respiration | Animal Heat |
|---|---|---|
| Before the exp. respiration | 128 | 104 |
| After | 52 | 102½ |
| 2 hours | 48 | 101½ |
| 4 | 52 | 102½ |
| 6 | 60 | 104½ |
| 8 | 52 | 95 |
| 8¾ | ceased | 90 |

*Experiment* 5.

| | Respiration | Animal Heat |
|---|---|---|
| Before the exp. respiration | 124 | 104 |
| After | 76 | 102½ |
| 2 hours | 80 | 102½ |
| 4 | 52 | 102½ |
| 6 | 60 | 101 |
| 8 | 56 | 95 |
| 10 | 48 | 88½ |
| 10½ | ceased | 86 |

*Experiment* 6.

| | Respiration | Animal Heat |
|---|---|---|
| Before the exp. respiration | 96 | 104 |
| After | 56 | 102½ |
| 2 hours | 68 | 102½ |
| 4 | 52 | 102½ |
| 6 | 72 | 104 |
| 8 | 52 | 105½ |
| 10 | 48 | 102 |
| 10¾ | ceased | 98" |

We do not understand this subject of animal heat. We could easily cite facts which seem to contradict each other. This apparent inconsistency is too often found, where the subtle operations of the nervous system are concerned.

offering the compliments of the season* to the time-honoured experimenter. May he have plenty of them. With his fund of good humour, and his professional zeal, they are certain to be not only merry, but useful ones, be they ever so many.

---

### STATISTICS OF REMEDIES IN DUBLIN.

Mr. W. Moore, Licentiate of Apothecaries' Hall in Dublin, has published in our valued contemporary of Ireland, a rather curious table. It is an analysis of the prescriptions received at the establishment opened by his grandfather in the year 1780. Accurate copies have been kept from that period, and they probably offer a rough view of the changes that have occurred in the practice of the most eminent physicians and surgeons of Dublin for a period of nearly sixty years.

"In order to effect this, I have divided the entire time into three equal portions; from each of these I have taken 1200 prescriptions, and marked the frequency with which each medicine occurred in them;—the results are shewn in the following table. The first column contains the years from 1780 to 98, the second terminates with the year 1817, and the third with 1836."

The following is the table.

| | | | |
|---|---|---|---|
| Emetics | 74 | 7 | 6 |
| Enemata | 58 | 7 | 20 |
| Blisters | 47 | 64 | 39 |
| Warm plasters | 45 | 10 | 3 |
| Leeches | 1 | 20 | 45 |
| *Antimonial Preparations.* | | | |
| Tartar emetic | 104 | 31 | 50 |
| James's powder | 4 | 36 | 44 |
| Kermes mineral | 8 | 3 | 0 |
| Antimonial powder | 14 | 28 | 15 |
| *Narcotics and Sedatives.* | | | |
| Opium | 102 | 79 | 116 |
| Morphia | 0 | 0 | 4 |
| Paregoric elixir | 72 | 28 | 13 |
| Digitalis | 0 | 8 | 3 |
| Hyoscyamus | 0 | 4 | 44 |
| Lactucarium | 0 | 0 | 11 |
| Peruvian Bark | 102 | 42 | 13 |
| Quinine, sulphate | 0 | 0 | 20 |
| *Saline Preparations.* | | | |
| Alum | 5 | 1 | 1 |
| Nitre | 45 | 9 | 11 |
| Glauber's salts | 35 | 12 | 2 |
| Sal polychrest | 38 | 0 | 0 |
| Sulphate of potash | 0 | 12 | 22 |
| Rochelle salt | 63 | 13 | 22 |
| Epsom salt | 0 | 213 | 102 |
| *Diaphoretics.* | | | |
| Mindererus's spirit | 67 | 35 | 22 |
| Nitrate of soda | 3 | 12 | 2 |
| ——— potash | 6 | 0 | 1 |
| ——— ammonia | 0 | 2 | 21 |
| Guaiacum | 32 | 10 | 2 |
| Sarsaparilla | 8 | 15 | 5 |
| *Stimulants, Antispasmodics, &c.* | | | |
| Castor | 8 | 10 | 1 |
| Musk | 4 | 1 | 0 |
| Assafœtida | 18 | 7 | 12 |
| Camphor | 9 | 22 | 52 |
| Ammonia | 64 | 88 | 39 |
| Ether | 9 | 22 | 2 |
| Spirit of nitrous ether | 20 | 10 | 10 |
| Iron | 22 | 42 | 20 |
| *Preparations of Mercury.* | | | |
| Calomel | 84 | 189 | 114 |
| Blue pill | 17 | 39 | 156 |
| Mercurial ointment | 35 | 11 | 1 |
| Ethiop's mineral | 15 | 0 | 0 |
| Mercury with chalk } Mercurius alcalizatus | 18 | 6 | 35 |
| *Purgatives and Aperients.* | | | |
| Jalap | 136 | 35 | 14 |
| Scammony | 80 | 120 | 51 |
| Colocynth | 19 | 31 | 67 |
| Aloes | 46 | 79 | 63 |
| Senna | 94 | 107 | 134 |
| Castor oil | 62 | 73 | 32 |
| Rhubarb | 81 | 59 | 170 |
| Magnesia | 70 | 43 | 52 |
| Syr. rosar. solutio | 21 | 0 | 0 |
| *Expectorants, &c.* | | | |
| Hippo | 62 | 58 | 152 |
| Ammoniacum | 21 | 26 | 11 |
| Squills | 33 | 47 | 45 |
| Iceland moss | 0 | 2 | 7 |
| Basilicon ointment | 32 | 7 | 0 |

*Dub.-Journ. September* 1836.

Mr. Moore adds some remarks on

---

* It is Christmas when we publish this.

this really interesting table. We shall only notice those of most consequence.

The enormous decrease in the exhibition of emetics appears to arise from the decline of the Cullenian doctrine of fever.

Enemata, almost lost in the second period, are again more frequently employed. They ceased to be extensively administered, when Dr. Hamilton drew attention to the value of purgatives given by the mouth.

Local bleeding has become much more popular. Broussais has probably contributed to this.

Opium has fairly kept its ground. The camphorated tincture has fallen.

Blue pill has steadily risen into favour. Mercurial inunction has as steadily declined.

The facts, in general, speak for themselves, and we recommend each reader to cast his eye attentively upon the table, and muse on the revolutions of drugs as of more mighty things.

---

### Abortion with Tardy Expulsion of an undecomposed Fœtus.*

Dr. Isaac Porter, of New London, Connecticut, has related the following case, as a practical reply to a query in a number of this Journal. In an article on the "Signs of Pregnancy," our reviewer had enquired :—

"How does atmospheric air get at blood confined within the uterus? We believe there is a medico-legal question now pending—perhaps decided—respecting the putrefaction, or supposed putrefaction, of the dead fœtus in utero. Does the fœtus become decomposed in utero when deprived of life?" Dr. Porter, we say, relates the following case in answer to this interrogatory.

*Case.* Mrs. ——— had proceeded to the fourth or fifth month of pregnancy, without experiencing any unusual circumstance. The signs of quickening, however, though anxiously expected, did not appear. With this exception, and the occurrence of severe dropsical symptoms, the other signs of pregnancy continued undiminished for another month. At this time the size of the abdomen began gradually to diminish, and at the eighth and ninth months was scarcely more prominent than ordinary. Still, a foreign body could, at times, be perceived through the parietes of the abdomen and uterus. The breasts also were distended, and there were occasional oozings of milk, though after the beginning of the ninth month, there was an evident diminution in the size and fulness of the former, and an almost entire interruption to the flow of the latter. The general health remained perfectly good. At the close of the ordinary period of utero-gestation, without pain, or any uncommon efforts, the distended membranes were found slightly protruding from the vagina—pains resembling cramp succeeded, and subsequently alarming hæmorrhage. Enveloped in the unbroken membranes, was a fœtus apparently of five months, which, upon delivery, was found free from any marks of decomposition. The placenta, which followed spontaneously, was in a morbid condition, being larger than ordinary, and resembling in form and consistence a sarcomatous tumor. At the usual period after delivery, milk was secreted in large quantity, and recovery was rapid and complete.

This case certainly appears to prove that a dead fœtus may reside for a considerable time within the uterus, without undergoing decomposition. The features of the case are all in favour of this conclusion.

"It may be mentioned, as a striking coincidence, that eight cases of abortion have come to the knowledge of the writer, which occurred during the month within which the death of the fœtus, in the case detailed, is supposed to have taken place, and all in this immediate neighbourhood. In these cases there was incipient putrefaction at the time the fœtus was expelled."

Our contemporary reports two cases, communicated to the Academy of Me-

---

* Amer. Jour. of Med. Sciences, Feb. 1836.

dicine on the 7th of March, 1835, by M. Vassal, in which a decomposed fœtus was expelled. In one case, the symptoms indicated gestation of four months, although the fœtus seemed one of about three months. When the membranes were ruptured was by no means clear. The mother recovered. In the second case, the woman was seized, at the end of the fifth month, with malaise, rigors, &c. She went her full time, when labour commenced, the membranes were ruptured, and a most abominably fetid, black fluid was discharged. The fœtus wanted the right arm, but the humerus, radius, and ulna were found in the bed in the coagula.

---

### SOME OBSERVATIONS ON THE USE OF THE HYDRIODATE OF POTASS AS A REMEDY FOR VENEREAL AND CACHECTIC SYMPTOMS.

There are few of our readers, who are not aware of the tendency to fashion that prevails in the exhibition of remedies. One year, calomel and opium are the rage—the next is consecrated to the glorification of colchicum—then every body must swallow strychnine—now, or, at least lately, iodine was the mode. Some men fall more easily into the periodical prescribing of strange remedies than others do. A man of good common sense is loth to give up the use of a medicine he is acquainted with, for that of one he knows nothing about. If an old remedy answers in a certain case, he naturally asks himself —"why should I change it? I know what it will do; but I do not know what effects this new drug may produce. When the remedies I am in the habit of employing fail, when the indications of treatment are not likely to be responded to by their familiar and well established properties, then I should be wrong not to try rational experiments with new medicines. Till then you must excuse my rushing headlong, to the hazard of my patient and my own disgrace, into senseless though shewy trials of a pharmacopœia of novelties." Such is the calm rebuke, we may imagine a physician or surgeon possessed of sound judgment and experience, offering to the rash prescriber of new drugs.

There are many reasons why such drugs should be abused. The love of novelty is always operative, and constitutes at once a very useful and a very dangerous element in human nature. It is useful because it is the source of all great inventions—it is dangerous, when not moderated by caution and good sense, because it leads to perpetual change.

This passion for novelty is backed at the present day, by the spirit of journalism. The rapid phases of the weekly prints offer temptations to men of strong desires and weak minds, to publish incessantly any thing new, no matter whether it be philosophically true or not. It is painful to be obliged to admit that the medical press is in an unsound condition. Reputations are too often sacrificed from a spirit of personal hostility, and as openly awarded from motives of personal favouritism. The professional merits of a man are determined by a standard, decisive enough, but certainly not honest. Does he belong to a certain party? If he does, he is a second Hunter, or a Baillie, or a Cuvier—an admirable lecturer, although he stutters to snoring pupils—an excellent practitioner, although every body of discretion is glad to escape from his clutches. If he does not belong to the proper party, his ignorance and imbecility become immediately conspicuous.

Party rage was always deemed to have gone its utmost lengths in the political world, and in the political press. Of late years, that press has been distinguished on the whole both for its ability and honesty. It is true, that extreme opinions have been advocated. Yet in the leading journals while political creeds are enforced with all the power of language, their columns are seldom polluted with gross ribaldry or disgusting personalities, never with deliberate untruths. It has been reserved for a scientific profession to exhibit in its literary appetite a keen relish for slander, and a taste for what is frequently neither more nor less than falsehood.

Journalism has encouraged the disposition to attract the profession, we will not say to humbug it, by positive assertions of the powers of new drugs. It is easy, as experience has proved, to get up a glittering array of cases, in which some newly-imported remedy has effected marvellous cures. The vulgar stare, and think that he must be a very clever fellow who has done all this. Gradually indeed the credulous are undeceived, and experience, after a season, shews how exaggerated the statements were. But the experimenter's purpose has been served. He has attracted notice, has been consulted in strange and in common cases, and as soon as the old bubble has fairly burst, he blows another. Such is a brief sketch of what has occurred on the professional stage. There have been too many actors in the farce, but we do not allude in particular to any one. Yet conscious sensitiveness may induce this or that individual to imagine, that we point at him.

> When you mention vice or bribe,

> 'Tis so pat to all the tribe :—

> Each cries—" that was levelled at me."

In the present article, we merely intend adverting to the hydriodate of potass, a remedy which has been latterly used in many complaints of very opposite descriptions, but has been especially employed in the treatment of venereal symptoms. We shall make a chapter in the work of Mr. Judd, and a lecture by Mr. Wallace, of Dublin, the text on which we shall hang some comments.

We have already drawn attention to the exhibition of the hydriodate of potass in cases of syphilis, particularly in two notices of two papers by Dr. Williams.* We remarked that, on the whole, the evidence adduced was unsatisfactory, and that the actual powers of the hydriodate yet remained to be determined.

Mr. Judd's observations on the hydriodate are imperfect. They are rather anticipations of what it may do, than actual statements of what it has done. The facts he states are four in number, and all are instances of secondary symptoms. We shall give a sketch of them.

1. A man had connexion with a prostitute on the 17th of June, 1833.

On the 20th, he had urethritis and a pimple on the body of the penis. On the 22nd, the pimple had become a sore, which healed on the 30th, and the urethritis ceased.

On the 27th of September, we find him with ecthyma, ulceration on the lower part of the tibia, an ulcer at the back of the throat, a gleet, and an excoriated glans penis. Under the employment of antimony, the warm bath, &c. followed by the infusion of gentian, the ecthyma declined, the ulceration healed, and chocolate-coloured stains, with œdematous ankles, and a few fresh flat pustules were left on the 28th of October. On the 1st of November there came out an eruption of lichen, which assumed a pustular character, and faded. The patient grew fat, but was pale and bloated, and suffered from severe nocturnal pains in his shins, when, on the 12th of December, he was ordered :—

*Potass. hydriod.* gr. v. *ter die.*

By the 24th, he was quite free from pains, and was in good health. In February, he had, for a fortnight, effusion into the abdomen, which was removed by purgative diuretics ; and by a report, date July 12, we find that there had been no relapse.

2. On the 10th of November, 1832, a man had connexion with a common woman. On the 2nd of January, a slight sore commenced on the glans penis, for which he took five grains of blue pill twice daily. How long this was continued it is difficult to say, for the case, displays the ordinary obscurity of Mr. Judd. On the 9th, bubo which did not suppurate. On the 12th the sore healed. On the 11th of February, after exposure to wet and cold, a sore throat succeeded by pains, and in April, by a discharge of pus and blood from the nostrils. On the 18th of May, he

* One of those papers was published in the Medical Gazette—the other in the first number of the St. Thomas's Hospital Reports.

presented rupia. It would appear that mercury was persevered in till the 19th, a period of upwards of four months! He suffered, with slight intermissions, from severe pains in the forehead and temple, the bloody discharge, now sanious, continuing. On the 24th of August, he was ordered to rub in, with temporary benefit. On the 18th of September, an abscess formed over the right os nasi, where a large ulcer formed. On the 4th of October there came pain and swelling of the left eyebrow, which was extremely indurated. Then followed an abscess in the lid, whence the probe passed to carious os frontis. On the 9th of November, periostitis of the left shin. From Aug. 30th to Nov. 11th, the mouth was kept sore. On the 19th, the protuberant part of the lid sloughed. On the 24th a large thin shell of bone came away from the throat.

26th. " The left nostril has become ulcerated within, and excoriated without; every now and then yellow discharge concretes upon it, and the patient is excessively plagued by it. A bent probe passed in by one nostril goes through the septum and out at the other; the part, once the bridge, of his nose has sunk upon the face so as to form a slightly curved hollow: he has a pallid leaden hue of countenance, he loses strength, and his nocturnal pains continue."

Thus he went on, his health being much impaired, his sufferings great, the nocturnal pains severe, the ankles swelling, when, on the 15th of December, he was ordered *potass. hydrio.* gr. v. *ter die in aquâ menth.*

To this was added a pint of new ale daily.

On the 21st, the nocturnal pains had almost ceased, and his health was better. On the 26th, the swelling of the forehead and eyelid had quite subsided. The ulcers soon after healed, and, in short, recovery took place in a highly satisfactory manner.

3. " In another secondary case, that of a young gentleman (the original disease not being contracted in England), with eruptions on the skin, pains in the shins and effusion into the synovial membrane of the knee, I easily effected a cure by this same remedy. He hunts and gets wet, and although more than a year has elapsed, he has not experienced any return of his complaint."

4. " The Hydriodate in the present month, in another instance, quickly removed an eruption and nocturnal pains; but the bubo remains to be healed. I am also trying the remedy upon a node, but have not continued its use long enough to ascertain how far it is efficacious in removing osseous depositions."

Such are the effects, such the evidence adduced by Mr. Judd. When we examine it we perceive that the cases in which the hydriodate was beneficial were of the cachectic character—cases to which mercury is notoriously, as a specific, inapplicable—cases, indeed, which are generally the result of the abuse of mercury. In the second case this was plainly the fact, and the unfortunate patient had good reason to complain of the treatment adopted in the first instance.

Let us turn to Mr. Wallace.

This gentleman has tried the effects of the hydriodate in many cases of secondary symptoms. He has formerly, it would seem, employed other preparations of iodine, but latterly he has confined himself exclusively to the hydriodate, and for rather a singular reason. Most persons would use this or that form of a medicine because it answered better than another. The chief consideration, however, with Mr. Wallace would appear to be a speculative one; at all events, he puts his hypothesis in the front of the battle.

" In the first place," he says, " I believe, that whatever preparation of iodine you use, it is only in the state of hydriodic acid, or of a hydriodate, that it enters the system. I have never been able to detect free iodine in any part of the body, or in any of the excretions. If you administer iodine in its free state to a dog, and examine the contents of his stomach, after the lapse of a very short time, you will not be able to detect an atom of iodine in the free state. You will find that it has all been converted into hydriodic acid. On one occasion Dr. O'Shaugnessy could detect no iodine in the matter discharged from the stomach of a dog to which he had given this substance fifteen minutes previously, but he found a large quantity of hydriodic acid. Are we not, from these facts, entitled to arrive at the conclusion that iodine, as I have said, enters the system only as an hydriodate or as hydriodic acid? If this be the case, it is quite useless to employ iodine in any other form, when it is our object to introduce it into the system."

We would observe that it by no means follows, because iodine is converted in the stomach into hydriodic acid, that therefore it is of no use giving iodine in any other form than that of hydriodic acid. This is as palpable a bull as ever gored paddy. If Mr. Wallace could shew that hydriodic acid was the only efficient form of the medicine, and that iodine exhibited in any other way would *not* be converted into hydriodic acid in the stomach, then his conclusion would logically follow from his premiss. As it is, we have a true Hybernian non sequitur. Put it in the form of a syllogism, and see how it looks. Hydriodic acid is the only form that gets into the system—but iodine, introduced into the stomach, immediately becomes hydriodic acid—therefore it is useless to give iodine.

The second reason which Mr. Wallace urges against any form of iodine but the hydriodate is a tangible one. He affirms first that iodine in its free state is very irritating to the stomach, the conversion into the hydriodic acid not being sufficiently rapid to prevent disturbance or even inflammation of the mucous membrane. And, secondly, he declares that the hydriodate of potass does not give rise to such injurious effects. This is the plain expression of a fact, and we have only to inquire if the fact is a true one.

We are disposed to go some way with Mr. Wallace, but not to the very end of his conclusion. We decidedly think that he overrates the mischievousness of the tincture of iodine, or of other forms of it besides the hydriodate. We have seen the tincture exhibited on a considerable scale, and we have given it freely ourselves. When the case was judiciously selected, and the medicine carefully managed, we have seen no bad effects whatever from its use. Perhaps there are few remedies which have been more extensively prescribed in London within these last few years, and though rash and ignorant people have no doubt abused it, still the experience of the best physicians and surgeons of the metropolis will certainly not be found to correspond with Mr. Wallace's. It is rather remarkable that when iodine was first employed for the relief of secondary symptoms, it was this very calumniated tincture, that effected those rather surprising cures which attracted the notice of the profession. It is a common practice to "give a dog a bad name," but, scientifically speaking, we may question its propriety.

While we defend the tincture of iodine against the unmerited obloquy heaped on it, we agree with Mr. Wallace that, on the whole, the hydriodate of potass is the preferable preparation. It is milder in its effects, less variable in its operation, and while it does not stimulate the intestinal mucous membrane so much, it appears to exert less of that peculiar depressing influence, occasionally witnessed after the exhibition of the tincture. Common experience is seldom, in the long-run, false, in cases to the determination of which it is applicable. That experience has, in this country, given the general preference to the hydriodate of potass. It is probable that Mr. Wallace has too bad an opinion of the tincture of iodine, but in declaring the superiority of the hydriodate of potass, at all events in venereal affections, he echoes the prevailing sentiment.

Mr. Wallace dwells much on the presence of the hydriodate in the urine, and in some of the secretions. There is nothing in this to attract attention. It is only what occurs with hundreds of drugs as well as with articles of diet.

The effects which he has observed to follow the employment of the medicine may be briefly stated:—

1. Increase of appetite, strength, and spirits.

2. Increase of the excretions, and secretions; of the urine—the perspiration—the saliva—increased action of the bowels. None of the effects are constant, and even the reverse of any one of them may happen.

3. Occasionally the hydriodate disagrees with the stomach, occasioning dyspepsia, heartburn, and a sort of sore throat. These accidents, Mr. Wallace informs us, are controllable by quinine.

4. Some patients have complained of watchfulness, and of a feeling in the head, approaching to headache. A purgative, and intermission of the medicine, have been necessary.

5. In two patients, who were overdosed, there was sickness, soreness of throat, colicky pains, vomiting, and purging to a slight degree, frequent pulse, and exhaustion. These symptoms were relieved by laxatives.

6. Several patients have been attacked with a pleuritic pain. When bled, the blood was cupped.

7. On one occasion purpura decidedly resulted from it.

8. In a patient who took the hydriodate indiscreetly and excessively by his own direction, there occurred indigestion of the character already noticed, with severe headache, and a rapid and quivering pulse. "But the most remarkable of his complaints was a peculiar state of his eyes,—such a state as I have never seen before or since. The pupils were dilated, and both his eyes were in a state of incessant motion. These motions strongly resembled those of a child who has congenital cataract. He found himself quite unable to fix them on any object. He kept his hand constantly over them, as if to shade them from the light, yet he seemed to say that the light did not hurt them." He complained of constant headache. Soon after this, he was seized with paralysis on one side of the body, preceded by muscular tremblings. He continued in a dangerous state for some weeks, when he began to amend, and has a reasonable prospect of recovery.

Such is the substance of Mr. Wallace's remarks. They appear to us to be very just, although we think he is inclined to under-estimate the ill effects and overrate the good that spring from the use of the hydriodate. Be that as it may, it is obviously a matter of importance to understand rightly the operation and the action of any remedy, particularly of one of so powerful a character as the one before us.

Our own experience, so far as it goes, and latterly we have employed the hydriodate in many cases, agrees very nearly with that of Mr. Wallace. The experience of a hospital is frequently not borne out in private practice, the constitutions of the patients in one and the other presenting material differences. This may account for our finding the hydriodate disagree much more than Mr. Wallace seems to have done. The patients to whom we have given it have been mostly private patients—persons in the respectable classes in London. The subjects of his observations have been chiefly in and out patients of the infirmaries to which he is attached. We may well conceive that the stomach of a Patlander accustomed to regale on potatoes and whiskey, will bear stronger stuff in the way of physic, than will that of an English gentleman or tradesman. Every one accustomed to hospital and private practice in London, is aware of the difference of dose necessary in the administration of many medicines, and of the difference of effect observed in their patients in the former and the latter.

Up to a certain point, the hydriodate of potass, like the iodine itself, appears to be a stimulant, if not a tonic. Certain it is, that it quickens the pulse, often increases the appetite and strength, will even occasion embonpoint, and excites more or less the whole system. It resembles in this respect arsenic, which, it is well known, will operate as a tonic in certain doses, and in certain cases.

Yet the hydriodate cannot be deemed a mere tonic. The medicines of that class exhibit, on the whole, very different effects. If we watch, for example, the operation of bark, we find that it excites the action of the heart, and of the capillaries, increases the strength, and invigorates the system. If pushed to a certain point, the bad effects that follow are just what might be supposed to result from an excess of the same sort of action. Too much blood is made, and it is circulated with too much force—sanguineous congestions or local inflammations ensue—the whole system is oppressed and overloaded—but none of that peculiar depression occurs, which marks the influence of a poisonous agent, exerted on the nervous system, or displayed through its medium.

To call arsenic a tonic, if by tonic is to be understood a remedy like bark, is clearly an abuse of terms. Arsenic, in appropriate doses, will at first quicken the action of the heart, and excite the system much in the same way that a tonic does. Acting thus, it will even cure diseases for which bark is notoriously efficacious—neuralgia, or intermittent fever. But here the analogy abruptly stops. If the arsenic is still given, or if its dose is increased, a new train of symptoms are suddenly developed. Either inflammation of the gastro-intestinal mucous membrane supervenes, or a prostration occurs, that is evidently the result of a powerful poisonous agent. We have seen arsenic, given for disease of the tongue, agree very well for a certain time, and all at once the patient has been seized with tremblings, rapid and weak pulse, and deadly exhaustion. One patient died in this condition, and, on dissection, nothing decisive was found. There was a slight redness of the gastric mucous membrane, and that was all.

There appears to us to be a greater analogy between the action of iodine and arsenic, than between that of iodine and any other medicine. We say analogy, for their specific effects are materially different.

Iodine, at first, like arsenic, stimulates the system, and excites the action of the heart and of the capillaries.* If carried too far, its effects are not simply, like those of quina, excess of stimulation, but, again like those of arsenic, poisonous and specific. The gastro-intestinal mucous membrane may suffer, or it may not. That is rather a local and accidental consequence. But whether it does or not, peculiar symptoms ensue—nervous agitation—rapidity and weakness of the heart's action—muscular tremblings and debility—general depression—and even death. We have seen all these effects, short of actual destruction, from the exhibition of iodine, both for cachexia and for malignant growths.

The hydriodate of potass differs from iodine in possessing more of the simply stimulating, and less of the peculiar and poisonous property. It is a difference, however, of degree and not of kind. The cases related by Mr. Wallace, and some that we could bring forward are sufficient to shew that, although it may be given with more freedom than free iodine, the same description of bad effects must be apprehended and guarded against. We need not fear them so much, but we must not forget that they *may* come.

Practically speaking, the injurious consequences of the hydriodate are rather of the immediate than remote description—they flow from its primary and stimulating qualities; not from its consecutive depressing ones. The mucous membrane of the stomach is irritated and excited, and secretes more acid, and the irritation extends to the œsophagus and pharynx. We have found uneasiness of the stomach, pyrosis, heart-burn, and that peculiar constriction and heat in the throat alluded to by Mr. Wallace, very commonly produced by a continuance in the employment of the medicine. Occasionally we see still more distinctly the effects of the stimulant property of

* When we talk of the action of the capillaries, we do not wish to advocate any hypothetical notions of their functions. We use the term for want of a better, and refer merely to the operations of the capillary system, exhalation and secretion and absorption, without troubling ourselves about their causes.

the hydriodate. Inflammation of the pleura, headache dependent on cerebral congestion, or inflammation of the gastro-intestinal mucous membrane, may ensue. It is not at all uncommon for persons who are taking this medicine to be attacked with heat of skin, headache, full pulse, loaded tongue, in short, with decided pyrexia. Probably, if the remedy were continued under these circumstances, either some positive local inflammation would arise, or the poisonous effects of the hydriodate would be developed.

We think, then, there can be little doubt that the sensible effects of the hydriodate of potass are in the first instance of a stimulating and a tonic character. We say sensible effects, for there may be, and there probably are others, of a more refined and subtle description, which not being shewn by any tangible alterations of the functions, are not so readily appreciated.

Were we to believe some authors, the hydriodate is a medicine of great value in all secondary venereal symptoms. This does not agree with what we have seen, nor is it likely à priori to be true. Few things can be more opposed in all essential features, than a secondary papular or tubercular eruption, and the cachectic rupia or ecthyma. The affection, in the first instance, may be the direct and simple consequence of the syphilitic virus, the constitution of the patient is good, the symptoms are of a sthenic character, and mercury, if properly exhibited, effects a cure. In rupia or ecthyma all bespeaks cachexia. The vesicles or pustules form scabs, foul ulcers, or become phagedænic or sloughing sores—the periosteum or the bones may be affected—the patient is emaciated—debility is evident—and, mercury, the abuse of which has usually caused the symptoms, almost always aggravates them. It is not likely, we say, à priori, that any one remedy should suit equally such opposite states. It is not consistent with our common experience of medicines or of maladies, nor does it agree with what we have observed of the effects of the hydriodate itself.

The venereal cases in which we have seen most benefit from the hydriodate, have been chiefly those of a cachectic character—cases in which repeated courses of mercury have been given, and in which the patient either does not require or will not bear that remedy. There are usually pustular or vesicular eruptions, producing scabs and unhealthy, sloughing, or creeping ulcers—ulceration of the throat of a character similar to that upon the skin—nodes, caries of the bones—chronic inflammation of the testis and the cord. Such is the kind of case in which the hydriodate appears, upon the whole, to do most good. It is frequently surprising to see how rapidly, and even permanently, the patient will recover. He may have gone on for months, or even for years, with only temporary and delusive periods of amendment, when a course of the hydriodate may set him at once and effectually upon his legs.

We lately attended a gentleman with Mr. Davis, an intelligent surgeon of Hertford. The patient had been ill for two or three years. When we saw him, there was considerable tumefaction of the alæ nasi, with redness of the integument, and discharge from the nostrils. There were several cachectic ulcerations on the skin—ulceration of the soft palate and pharynx—emaciation—and general ill-health. We prescribed sarsaparilla and some other remedies, and the patient mended a little. But portions of diseased bone came away from the nose, and a piece, apparently of the horizontal process of the palate bone, was discharged through an ulcerated aperture in the front of the velum. We now put the patient on the hydriodate. The result was most gratifying. All the symptoms very speedily subsided, and the gentleman has been left with a small, round, circular aperture in the palate, productive of too little inconvenience to require the application of the suture.

We must own, that although occasionally the hydriodate has effected both rapid and permanent benefit, it has not in the majority of instances done so. It is the character of the cachectic disease we have mentioned, to return again and again, and to exhibit repeated vicissitudes of improvement and relapse. It is indeed a singular complaint, and one well calculated to illustrate the doctrines of the humoral pathology. A patient is attacked with an eruption of rupia or ecthyma, and the vesicles or pustules ulcerate or slough—at the same time, perhaps, ulceration of the throat appears or spreads, or sloughing invades the mucous membrane of the fauces or the nares. After a time these symptoms become mitigated—the sloughing is arrested—the ulcerations may heal—and the patient may even seem to be restored to health. But the recovery is deceptive. Suddenly, he is again seized with the whole or with a part of his former symptoms, which again exhibit the same successive phases of aggravation, arrest, and amendment or apparent cure. This scene may be, and generally is repeated many times, and the event depends on the treatment employed, and on the constitution of the patient. If both are bad, the attacks grow more and more serious, or rather they leave him less and less able to sustain them. The bones may or may not become affected, but, whatever be the character of the specific symptoms, it is not from them, per se, that death usually results. Accidental circumstances lead to disease of some vital organ, and, in the majority of instances, either phthisis, or fever ending in ulcerations of the mucous membrane of the bowels, terminates the case.

It could hardly be expected that any remedy would at once cut short a malady of such a character. It is clearly a constitutional disease, and, like scrofula, depends on too general an alteration of the system to be at once put out. Such anticipations are supported by experience, and it is seldom indeed that a case of any severity is quickly cured. Of course, there are differences of degree in this, as in all other complaints, but we repeat that we must not anticipate an off-hand *and* permanent recovery from any system of management, undoubtedly not from any single drug.

In many of these cases the hydriodate agrees remarkably well. Given at the proper time, it seems to arrest the phagedænic action, promote the cicatrization of the sores, and re-establish the health of the patient in a very remarkable degree. But many other remedies do the same thing. Sarsaparilla—the mineral acids—tonics—sometimes small doses of mercury, produce in these cases more or less of the same effects as the hydriodate. In short, it is preposterous to term this medicine a specific for this complaint, for though sometimes it answers better than any of the others we have mentioned, it not unfrequently does not answer so well. It is an addition, and a valuable addition to our means of treatment—it gives us one other medicine to ring the changes with, and that is the most that can fairly be said of it. We may mention a case which is sufficiently illustrative of these remarks.

*Case.* A respectable tradesman, after a long and injudicious course of mercury for a venereal sore, was attacked with extensive ulceration of the fauces, and with a phagedænic ulceration on the left arm, which succeeded a vesicle that scabbed. A variety of means were tried. Sometimes he was better, sometimes he became worse. He was put on the hydriodate in full doses, with almost instantaneous benefit. The ulcer of the throat almost healed. Again it broke out, and again the hydriodate effected the greatest benefit. But still these relapses occurred, and now, at the end of two years and a half from the first appearance of the ulceration in the throat, the patient is in a very precarious condition. The fauces are covered with a sloughy ulceration. A portion of the lateral and nearly all the vertical cartilage of the nose have been destroyed — and gastro-enteritis following the *cautious* employment of the hydriodate nearly proved fatal about a month ago. In each attack the medicine has appeared to be of signal service, yet two years and a half have passed, and the individual is not materially benefited, but rather the reverse.

We could mention many cases of this description, and we can assure our readers that there is not a greater error than to suppose that, because the hydriodate is of temporary, it is always of permanent service. It is a remedy which should be employed with judgment, caution, and that distrust which *must* spring from an acquaintance with the peculiar characters of the disease.

Not unfrequently, it is either of no service at all, or it positively disagrees.

We have at present under our care a gentleman, whose case has been in some respects a singular one. Having had, to his knowledge, neither sore nor urethral discharge, he was attacked with yellow ulceration of the throat, and what looked precisely like the venereal tubercle. He consulted a surgeon, who felt unwilling to order mercury, and prescribed the tincture of iodine. The ulceration quickly healed, and the eruption appeared to decline. But soon both re-appeared. We saw him about nine months after the commencement of the ulceration of the throat. Both tonsils were then the seat of sloughy ulcers, and there were several spots of rupia on the skin. He had also secondary ulcerations at the verge of the anus, which were exquisitely painful. We ordered a course of calomel and opium, under which the ulcerations of the rectum and of the throat healed, and have never since returned. But the rupia spots ulcerated, and at intervals fresh sloughy ulcerations have appeared on different parts of the body, accompanied latterly with nodes. We have repeatedly tried the hydriodate in this case. After a few days, it occasions heart-burn, pain at the epigastrium, pyrexia, and a more sloughy state of the sores. To these it has never been of service, and the patient has been always made ill by it.

In cachectic cases of a more obscure character, and totally unconnected with syphilis or with the abuse of mercury, the hydriodate occasionally does wonders.

*Case.* A gentleman was seized with what appeared to be pleurisy. He was bled to a great, indeed to an improper extent. He had previously been a free liver, and the depletion reduced him in such a manner, that he never properly rallied. Under these circumstances, he was attacked with violent pains in the bones—he was sallow, emaciated, and had all the appearance of a man broken down with the venereal cachexia. He was under the care of the most eminent physicians at Cheltenham, but got no better, or not materially so. He came to London, and consulted an eminent surgeon, who prescribed the hydriodate. The pains in a week or two disappeared, and he rapidly recovered. Whether the cure will be permanent, it is of course impossible to say.

The hydriodate has been applied to the treatment of other venereal affections, besides the cachexiæ we have spoken of. In the present article, we confine ourselves to what we have personally witnessed, and shall, therefore, state only the results of our own observation.

1. We have not tried it in cases of primary syphilis, nor do we *imagine*, from what we know of its operation, that it will be of service. Perhaps, in some cases of primary phagedænic ulceration, it may be useful.

2. Nor, from what we have witnessed, should we conceive that it is applicable to the pure syphilitic eruptions—the scaly, or the tubercular. These eruptions are so much under the control of mercury, and its action is so different from that of the hydriodate, that it seems both unnecessary and unreasonable to employ, or to anticipate much from the employment of the latter.

3. It has been lauded in cases of papular eruption. But we should pause before we attach much importance to its use in *them*. Lichen is so fugitive in its character, that it is difficult to determine the effects of remedies upon it—at all events, there is an obvious source of fallacy.

4. We have tried the hydriodate in some of the more vague secondary venereal symptoms.

*a.* In a case of enlarged tonsils, covered with white superficial ulceration, and connected with vaginal discharge and condyloma, it was of no benefit.

*b.* A gentleman had sores, which were said in Paris to be excoriations, and were treated in England as syphilitic.

He seems to have taken a good deal of mercury. His health had been impaired by previous dysentery, contracted in Syria, while travelling for amusement. Two years afterwards we saw him. He was liable to frequent irruptions of small superficial sores on the lips and tongue, and had psoriasis palmaria. We have tried the hydriodate three times in this gentleman. Each time it occasioned dyspepsia, and did no good to the complaint. In another similar case, we used the medicine with the same want of success. In a third case, it was of use, but the disorder was getting well, when we ordered the hydriodate to be taken.

Such are the results of our own experience. We have employed the hydriodate in other cases, but the results were not sufficiently distinct to induce us to allude to them. On the whole, we are disposed to coincide with those who have found the hydriodate answer best in cachectic cases. But we think that, even in these, it is uncertain, and that it has been overrated. It is another string, and not a bad one, to a bow that has several already, but requires more.

The most usual mode of exhibiting the hydriodate, is in the state of solution in camphor mixture. It is nauseous in that form. Perhaps the pleasantest formula is the succeeding, or one like it.

℞. Aquæ Florum Aurantii, ℨiij.

Aquæ Distill. . . . . . . . . ℥j.

Syrupi Aurantii . . . . . . ℨj.

Potass. Hydriodatis . . . gr. v. ad x.

M. ft Haustus ter die bibendus.

We have found that, in cachectic cases, it is usually better to conjoin the hydriodate with small quantities of sarsaparilla. Either the fluid vehicle of the hydriodate may be sweetened with a drachm or two of the syrup of sarsaparilla, or the latter, in the most appropriate form, may be taken a little after the dose of the hydriodate.

In private practice, patients will seldom bear more than ten or twelve grains of the hydriodate thrice daily. It is better to begin with small doses, and gradually increase them. We may commence with five grains twice in the day, and augment the proportion to the requisite amount.

---

### Ulceration of the Cæcum, and Abcess of the Right Iliac Fossa, communicating with the Interior of the Bladder, and producing the Symptoms of "Irritable Bladder." By Henry James Johnson.

The following case is in some respects so remarkable as to deserve to be recorded. The diseases of the urinary organs merit in a high degree the attention of the surgeon, for they are numerous, important, and not unfrequently obscure. It is only by a very careful examination of the urine, and by a comparison of its states with the other symptoms, that we can hope to guard ourselves against the many sources of error, that interfere with the formation of a correct opinion.

The facts that have been elicited by recent investigations, with respect to the pathological changes that accompany an albuminous condition of the urine, are of a highly interesting character. It has been proved, beyond the possibility of doubt, that such an alteration of the urine, if permanent, and particularly, if accompanied with prominent symptoms of disturbance of the bladder or the kidneys, is usually the consequence of organic changes in the latter. Of all the diseases of the kidney, and there are several, that lead to the secretion of albuminous urine, chronic inflammation, ending either in the mottled state described by Dr. Bright, or in abscesses in the cortical or tubular structure, is the most frequent.

It is singular to observe the variety displayed in the symptoms of what, for want of more exact information, we must consider chronic inflammation of the kidney. In one patient, the irritability of the bladder is excessive, and he seems to labour under all the miseries occasioned by the presence of a stone in it. In another patient, there is nothing which attracts his own, or, perhaps, the physician's attention to the urinary apparatus. The extent of lesion is no measure of the severity of symptoms, nor is severity of symptoms a positive indication of any lesion at all. I examined lately the body of a gentleman who died worn out by the cachexia induced by the abuse of mercury. His urine for many weeks before his death, was albuminous, and there was great irritability of the bladder. Yet, on dissection, I could detect no further disease in the kidney, than a very dubious injection of the mucous membrane of the pelvis of the ureter. Why one individual, affected with chronic inflammation of the kidney, should labour under severe symptoms of disturbance of the bladder—and why another, with equal or with greater renal disease, should not, are questions which it appears impossible to answer satisfactorily at present.

Contradictory as facts in some measure are, obscure as the symptoms of alterations of the kidney in their earlier stages must be owned in many cases to be, there are some phenomena which, taken in the aggregate, will not often deceive the acute and experienced observer.

When a patient complains of extreme frequency of making water—when he passes very small quantities at a time—when, in the act of micturition, and after it, he experiences a cutting pain at the end of the penis, and uneasiness referred to the perineum or to the neck of the bladder—when the urine is decidedly albuminous, acid, and free from mucus—and when, on sounding the bladder, no stone is discovered in it, while examination by the rectum detects no change in the former organ or urethra—the surgeon may suspect, and his opinion will be very seldom a false one, that the symptoms depend on disease of the kidney.

It is not my object to go into the history of chronic inflammation of the kidney. Our knowledge of the pathological change in its commencement—of the symptoms that then mark it, if any positive symptoms do so—and of the treatment of the malady in all stages—is, unfortunately, both limited and vague. My reason for making these observations has been to clear the way for the appreciation of the characters of the case that I now proceed to describe. It will be seen that the case presented such symptoms as will generally justify us, in supposing that renal disease exists, yet the event shewed that there was none.

*Case.* In the latter part of last Summer, I was requested to see a respectable young man, a tradesman, who was then in the following condition.

He was much emaciated, and had all the external appearances of serious organic disease.

What he principally suffered from was frequency of micturition. He was obliged to make water every half hour—he passed little more than an ounce at a time—prior to the act of micturition he experienced uneasiness in the region of the bladder, and dull pain in the perineum—in the act, he had cutting pain at the end of the penis—and this continued for some little time after the last drops of urine had been voided. He was not aware that the stream of urine was ever suddenly arrested, but once or twice there had been a tinge of blood in it, and the last drops were always discharged with tediousness and difficulty. He complained of no pain in the back, but referred to the urethra and the bladder as the principal seats of his uneasiness. Pressure over the pubes was productive of pain.

There was cough—the respiration was imperfect and hurried—he had at times what, from his description, appeared to be purulent expectoration—the chest sounded indifferently on percussion—there was bronchial respiration pretty generally in the upper lobe of each lung—and pectoriloquy existed under the right clavicle. Thus the general and the physical signs of phthisis were unequivocal. The pulse was frequent—there was a disposition to hectic—the bowels were rather irregular, being sometimes confined, and sometimes rather relaxed.

The urine was pale, rather turbid, and acid. On applying nitric acid and heat, the presence of albumen was distinctly shewn, but there was in addition a sort of precipitate of rather opaque whitish flocculi, which were not unlike adipocire in *appearance.**

On introducing a catheter into the bladder, I found that the patient was able to empty it in the ordinary act of micturition. No calculus could be detected. The introduction of the instrument was attended with much spasm of the muscles of the bulb, and with considerable irritation and pain in the perineum and bladder. Whilst the instrument was in the latter, the finger was introduced into the rectum, and pressure made on the prostate and trigone. This occasioned comparatively little inconvenience.

The history of the case may be briefly told. For some months the patient had laboured under phthisical symptoms, for which he had been judiciously treated by his usual medical attendant, Dr. Thompson. These symptoms were relieved, when, suddenly, about two months before he applied to me, he was attacked with a frequent desire to make water, and with the other symptoms of irritation of the bladder. The means employed to relieve the disorder were attended with little benefit, and he had gradually been growing worse before he came under my observation. It is proper to observe that from the period of the accession of the urinary complaint, the thoracic had declined in prominence and in importance.

As the usual symptoms of disease of the bladder were not present—as I could not detect the existence of a calculus—and as the urine was albuminous, I conceived that the disease was probably in the kidney, and that the irritability of the bladder was merely symptomatic of that. Yet the suddenness of the access of the symptoms, the anomalous deposit in the urine, and the uneasiness on pressure in the region of the bladder above the pubes, were circumstances that I could not satisfactorily explain.

I applied leeches to the hypogastrium, and prescribed a combination of the tincture of henbane with the liquor opii sedativus and the liquor potassæ. Under these means, combined with rest in the recumbent posture, a regulated diet, and attention to the bowels, the pain on pressure above the pubes disappeared, and the frequency of micturition was rather diminished.

Various means of lessening the irritability of the bladder were tried. All medicines possessed of a diuretic property were injurious. The liquor potassæ, the infusion of the uva ursi, the infusion of buchu, severally increased both the frequency of micturition and the attendant uneasiness and pain.—Narcotics, whether given by the mouth, or in the form of injection, or in that of suppository, were alone of any service. They reduced the times of making-water to four or five in the day, and about as many in the night, and the quantity of water passed at once was augmented to a quarter, or even to half a pint. The irritability of the bladder varied from day to day, without adequate apparent cause. One night the patient would only be compelled to rise three or four times, on the next, he would, perhaps, be disturbed nine or ten times. The quantity of albumen contained in the urine gradually decreased, although in this too there was much variation. On one day there would be a large albuminous precipitate, and two or three days afterwards, the application of acid would merely render the urine rather cloudy.

Although, upon the whole, the urinary symptoms were much mitigated, and the sufferings of the patient materially diminished, he evidently grew weaker. It was between two and three months from my first seeing him, when he rather suddenly died. This was in the commencement of October last. I examined the body, with the assistance of Dr. Thompson and of Mr. Oliver, one of my pupils.

* I regret that this was not more carefully examined. I had intended to have requested some gentleman practically conversant with analytical chemistry to ascertain the exact nature of this unusual deposit.

The emaciation had gone to a great extent.

There were tubercles in both lungs, to the upper lobes of which they were confined. Some of the tubercles were softening, and in the apex of the right lung were two or three small vomicæ.

The kidneys were perfectly sound, and the ureters were unobstructed.

The bladder appeared, at first sight, healthy. There was no inflammation of its mucous membrane, no thickening of its parietes. But on its anterior wall, about the junction of its middle and upper third, was a small, round, fistulous opening, scarcely of the diameter of a common pea. The mucous membrane on the margin of the aperture was perceptibly injected, and the aperture itself had been evidently the result of ulceration. The aperture in the bladder communicated with a sinus in the cellular membrane between the organ and the abdominal muscles at their pubic insertion. From this point the sinus proceeded in the cellular membrane between the muscles on the right side and the peritoneum to the right iliac fossa, where it opened into a sort of abscess, partly formed by the cellular membrane there, partly by the cæcum, a large portion of the wall of which was destroyed by ulceration.—Thus the bowel was freely laid open into the cellular membrane of the iliac fossa, from which a sort of winding abscess proceeded to communicate with the interior of the bladder. The mucous membrane of the cæcum, of the ileum at the ileo-colic valve, and of a small part of the ascending colon, was in a state of ulceration.

No other morbid appearance was discovered.

There can be little doubt of the real nature of this interesting case. It was originally one of ulceration of the mucous membrane of the cæcum, that affection being perhaps connected with the softening of pulmonary tubercles.* The ulceration extending through all the coats of the bowel, its interior was laid open into the cellular membrane of the iliac fossa, and a fæcal abscess resulted. The suppuration gradually travelled along the continuous cellular membrane, until it arrived at the front of the bladder. In this it occasioned ulceration, and the fistulous aperture that followed. It is probable that the sudden access of symptoms of irritation of the bladder, marked the period of its perforation.

Why the abscess should take this course, and why the aperture in the bladder should be so small, it would now be useless to inquire. It is clear that the albumen contained in the urine, was occasioned by the pus that found its way into the bladder.

But it is not so easy to determine the immediate cause of the irritability of that organ. Was it the existence of the small fistulous opening in it, or was it the discharge admitted into the bladder through the opening? The irritability of this organ, which results from disease of the kidney itself, is exceedingly capricious. Sometimes there is considerable disorganization of the kidney, and the urine is highly albuminous, without any, or with very little irritation of the bladder—sometimes this is excessive, with only moderate alteration of the kidney, and with only a small quantity of albumen in the urine. It is obvious that the careful observation of facts, and the accurate discrimination of particulars can alone explain these apparent inconsistencies, if they admit of satisfactory explanation at all.

---

### Dr. M'Cabe on Diseases connected with Spiculæ of Bone in the Skull.

*Hastings, Nov.* 10, 1836.

Gentlemen,

In the last Number (50) of your invaluable Journal, I read the heart-

* Ulceration of the mucous membrane of the termination of the ileum and commencement of the great intestine, is common in advanced phthisis, and is seen occasionally in *its* incipient stage.

sickening detail of the sufferings and fatal termination from chorea, of Miss ——, your interesting patient.

In describing the post-mortem appearances in this case you observe, "it will probably be doubted by many, whether such frightful phenomena can be fairly attributed to such small matters as two sharp points of bone, on the internal surface of the skull," &c. It is true that certainty on this point cannot be attained; but, reasoning from the symptoms during life, and the appearances after death, *in two cases* which came under my care, I feel little doubt in agreeing in your observation—" that it was very improbable (had you been permitted to examine the spinal marrow) that any thing further would have been found to account for the terrible symptoms" it was your painful duty to witness.

*Case* 1. About five years ago, in my attendance upon a young gentleman, I had frequent opportunities of seeing his father, Mr. F——d, then about 40 years of age, who, as I understood from his lady, had six years previously been in excellent health—highly talented, and a valuable member of society; about the time mentioned, and without any apparent cause whatever, he perceived his vision gradually impaired, with (from time to time) severe head-aches about the forehead. His vision, however, rather improved, but in a short time symptoms of mental derangement supervened, for which he went through the regular routine of the practice of various mad doctors, until he settled into a state of idiotcy, with occasional fits of troublesome excitement:—At length a fit of apoplexy occurred, which in about a week terminated fatally.

Assisted by my friends, Dr. Chapman and Mr. Ranking, the head was carefully examined. To our surprise, we observed the two anterior clinoid processes of the sphenoid bone much longer than natural; one of them a full quarter of an inch in length, and sharp as the point of a fine penknife—the other, although not quite so long, shewing the same disposition to morbid growth; the soft substance of the brain, into which these points penetrated, was inflamed and very soft—but there was very little effusion into the ventricles or between the membranes.

*Case* 2. Two years since, in conjunction with (I believe) the above-named gentlemen, I inspected the head of a young lady, Miss T——r, of great moral worth and highly talented. For many years she had been subject to tormenting headaches, principally about the occiput: in one of these attacks, alarming symptoms of phrenitis supervened, which baffled all I knew of remedial means. Great nervous exhaustion and coma followed, which terminated the sufferings of this amiable person, in about 12 days from the commencement of the attack.

At the base of the skull, on cutting into a portion of the brain, the scalpel met a hard substance, which proved to be a spicula of bone, a full quarter (or more) of an inch in length, but not perpendicular—rather inclined, with a double jagged point, not unlike the junction of the two points of a forceps. As in the former case, the neighbouring part of the brain was soft and inflamed, and, like the other case, very little effusion into the ventricles, or between the membranes.

---

### Semi-Poisoning from Iodine.

In my humble sphere of practice, I have, as regards new medicines, generally attended to the advice, as regards fashion in dress—

> " Be not the *first* by whom the new is tryed,
> Nor yet the *last* to lay the old aside."

I have seen the following results from the exhibition of iodine.

A poor lad, aged 14, with considerable swellings of the glands of the neck, was brought to me from the country for my advice. I prescribed for him *five* grains of iodine and fifteen of the hyd. potassæ in an ounce of distilled water; fifteen drops of the solution to be taken *three* times a-day, in a wine-glass of water—also an ointment, of about the same quantity of the above medicine to six drachms of lard, the size of a nut, to be rubbed on the glands every night. As the boy lived fifteen miles from Hastings, I did not see him for a fortnight; at that interval he again came to me—I found his appearance very much changed for the worse; from a stout healthy-looking boy, he became thin and emaciated—the swellings of the neck were certainly diminished, but his whole body was covered with large livid petechiæ, and his mother informed me that, for the last three or four nights, he had bled profusely from the mouth and nose during sleep, the blood being, however, very pale and thin. I of course ordered the medicine to be discontinued, and directed for him an astringent mixture of the infusion of roses, with an additional quantity of acid, and furnished him with a bottle of port-wine—the first for the credit of pharmacy, the latter for good fellowship, and to repair, in some measure, the mischief I had caused to the poor fellow. Under *these* remedies, he got soon well of the constitutional symptoms, leaving me to consider other means for the reduction of the "embonpoint" of the neck.

I am, Gentlemen,
Your obedient Servant,
P. M'CABE, M.D.

---

SEVERE INJURY OF THE BRAIN—SUPPURATION—TREPHINE—INCISION OF DURA MATER—RECOVERY. By Mr. R. M. FORSAYETH, of Templemore, Tipperary.

On the 21st of August, 1836, I visited Henry Oldfield, aged 18, who, eleven days before, received a blow from a reaping-hook on the upper and back part of the frontal bone, penetrating it, the dura mater, and brain. Had *very slight* paralysis of opposite side—some pain in the head—total loss of speech—other senses perfect: pus oozes through the hole. Introducing a probe gently, it entered nearly two inches, causing it to flow freer, and giving entire ease to pain: pupils regular—pulse 56—has no other complaint whatever; was bled and had medicines before I saw him. I laid bare the fracture; the external table was plainly driven into the diploë, a curious burr surrounding the hole in the skull. To have calomel, gr. iij. and opium ¼, 4to die, till the gums were touched; diet light. Gave directions to his father, an intelligent man, to watch the effect of the medicine, and let me know how he went on, particularly if the hole filled up.

He seemed improving fast until the 28th, when I saw him again, kindly accompanied by Dr. Dix, of the 94th regiment. He got very heavy: all discharge stopped by a fungus from the diploë: on introducing a probe, pus followed. I applied the trephine, and took out a piece of very thick skull; there was a small quantity of coagulated blood on the dura mater, which was rather redder than usual—also a hole corresponding to that in the skull, and a circular piece of bone of the internal table, that gave way before the hook, and slipped to one side. Pus did not flow from the hole in the dura mater, but it followed the probe, introduced an inch or so, and ceased on withdrawing it, as if a valve lay at the opening. Dr. Dix suggested the propriety, at all hazards, of opening the dura mater. I made two *deep incisions* in it, from side to side of the trephined space, and obtained altogether about ℥ss of pus—put on soft dressing, and left him. He, a quarter of an hour afterwards, felt much lighter—an occasional dose of the calomel to be still given. The discharge lessened daily; the wound healed kindly, yet slowly, and he is now quite well—a manifest, though slow improvement in the speech.

I had an exactly similar fatal case (except the speech) where the piece of bone was carried into the brain, causing an abscess three inches deep. The boy was trephined—lived six weeks, and died from the wound closing, and confining the discharge. After this case, I would not have hesitated to make a deep incision into the brain, and search, cautiously indeed, for the bit of bone.

